# Periodontitis and systemic inflammation as independent and interacting risk factors for mortality: evidence from a prospective cohort study

**DOI:** 10.1186/s12916-023-03139-4

**Published:** 2023-11-13

**Authors:** Christiane Pink, Birte Holtfreter, Henry Völzke, Matthias Nauck, Marcus Dörr, Thomas Kocher

**Affiliations:** 1 Department of Restorative Dentistry, Periodontology, Endodontology and Preventive and Pediatric Dentistry, University Medicine Greifswald, Fleischmannstr. 42, 17475 Greifswald, Germany; 2 Department of Orthodontics, University Medicine Greifswald, Greifswald, Germany; 3 German Centre for Cardiovascular Research (DZHK), Partner Site Greifswald, Greifswald, Germany; 4 Institute for Community Medicine, SHIP/Clinical-Epidemiological Research, University Medicine Greifswald, Greifswald, Germany; 5 Institute of Clinical Chemistry and Laboratory Medicine, University Medicine Greifswald, Greifswald, Germany; 6 Department of Internal Medicine B, University Medicine Greifswald, Greifswald, Germany

**Keywords:** Periodontitis, Systemic inflammation, Cardiovascular disease, Mortality risk, Interaction, Survival analyses, Cohort study, Study of Health in Pomerania

## Abstract

**Background:**

Recent studies have highlighted the role of low-grade systemic inflammation in linking periodontitis to cardiovascular disease (CVD) outcomes, but many aspects remain unclear. This study examines the independent and reciprocal associations of periodontitis and low-grade systemic inflammation with all-cause and CVD mortality in a large-scale cohort.

**Methods:**

A total of 3047 participants from the prospective, population-based Study of Health in Pomerania (SHIP-START) were followed for a period of 13.0 ± 2.4 years. For the association between various inflammation/periodontitis measures and mortality, hazard ratios (HRs) were obtained from covariate-adjusted Cox proportional hazards models. Interactions were analysed in joint models: on the multiplicative scale, HRs were reported and on the additive scale, relative excess risks due to interaction (RERI) were calculated. Subject and variable-specific interval records were used to account for time-varying exposures and covariates.

**Results:**

During the observation period, 380 (12.5%) individuals died from CVD (*n* = 125) or other causes (*n* = 255). All markers of periodontitis and inflammation showed apparent associations with all-cause mortality (HRs per SD-increase: mean PPD: 1.068 (95% confidence interval (CI): 0.988–1.155), mean CAL: 1.205 (95% CI: 1.097–1.323), missing teeth: 1.180 (95% CI: 1.065–1.307), periodontitis score: 1.394 (95% CI: 1.202–1.616), leukocytes: 1.264 (95% CI: 1.163–1.374), fibrinogen: 1.120 (95% CI: 1.030–1.218), CRP: 1.231 (95% CI: 1.109–1.366), inflammation score: 1.358 (95% CI: 1.210–1.523)). For CVD mortality, all PPD related variables showed significant associations. Interaction modelling revealed some variation with respect to mortality type and exposure combinations. On the additive scale, RERIs for periodontitis score and inflammation score implied 18.9% and 27.8% excess mortality risk for all-cause and CVD mortality, respectively. On the multiplicative scale, the HRs for interaction were marginal.

**Conclusions:**

Both periodontitis and inflammation were significantly associated with all-cause mortality and CVD mortality. On the additive scale, a substantial excess risk was observed due to the interaction of periodontitis and inflammation, suggesting that the greatest treatment benefit may be achieved in patients with both periodontitis and high systemic inflammation. As periodontal therapy has been reported to also reduce systemic inflammation, the possibility of a reduction in CVD mortality risk by anti-inflammatory treatments, including periodontal interventions, seems worthy of further investigation.

**Supplementary Information:**

The online version contains supplementary material available at 10.1186/s12916-023-03139-4.

## Background

Cardiovascular disease (CVD) has been one of the leading causes of death for several years and continues to increase in prevalence and mortality. According to a recent World Health Organization (WHO) statement, ischaemic heart disease and stroke were responsible for about 16% and 11% of all deaths worldwide in 2019, making them the leading causes of death [1]. These thrombotic complications of atherosclerosis are closely linked to systemic inflammation, as a variety of inflammatory mediators play a critical role in all stages of the atherosclerotic process [2–4]. Indeed, recent results from large clinical trials have shown promising results for anti-inflammatory drugs in reducing CVD events and mortality in patients with atherosclerosis [5–7].

Systemic inflammation is also closely associated with periodontitis, a bacterially induced chronic inflammation of the tooth-supporting tissues [8]. Periodontitis contributes to systemic inflammation by promoting bacteraemia during personal oral hygiene, chewing or dental treatment [9, 10] and also by the continuous spillover of locally produced inflammatory mediators from the compromised gingival tissue into the bloodstream [11, 12].

In conjunction with certain genetic components [13], a large body of evidence supports the role of systemic inflammation as an important mechanistic link between periodontitis and CVD [14, 15]. People with periodontitis have a higher prevalence of subclinical CVD as well as an increased risk of myocardial infarction, stroke and further adverse events [16]. With a global age-standardised prevalence of 11.2%, severe periodontitis is one of the most common human disorders today [17]. Thus, the relationship between periodontitis and CVD, particularly CVD events and mortality, may be of concern to a substantial number of people worldwide. However, many aspects of this association are not yet fully understood [18]. Further research is needed to assess the extent to which reducing periodontal inflammation may influence mortality risk by reducing the overall systemic inflammatory state.

Therefore, the primary objective of the present study was to investigate the extent to which periodontitis and systemic inflammation are longitudinally associated with mortality in the population-based Study of Health in Pomerania (SHIP-START). This includes independent effects of periodontitis and systemic inflammation but mainly focuses on their interaction and the resulting effects on all-cause and CVD mortality risk. Different measures of both exposures are presented, compared and combined, taking into account the possibility of additive and multiplicative interactions as well as effect mediation.

## Methods

### Study population

SHIP-START is an ongoing population-based prospective cohort study in the north-eastern region of Germany. A two-stage stratified cluster design was applied to sample Caucasian individuals with primary residence in the study area. First, three cities, 12 larger towns and 17 surrounding villages were selected. Second, a sample of 7006 subjects, stratified by age and sex, was drawn from the population registers in proportion to the size of each municipality. Excluding 741 neutral losses (126 deaths and 615 migrations), 4308 of the 6265 eligible subjects finally participated in the baseline examinations (SHIP-START-0) between 1997 and 2001 (response rate 68.8%). Follow-up examinations were conducted from 2002 to 2006 (5-year follow-up, SHIP-START-1) and again from 2008 to 2012 (11-year follow-up, SHIP-START-2). All SHIP waves included questionnaires and interviews on lifestyle, health and risk factors, as well as comprehensive medical and dental examinations. Participants gave written informed consent and the study protocol was approved a priori by the local Ethical Review Board. Further details of the SHIP study design and protocol have already been published elsewhere [19].

**Fig. 1:**
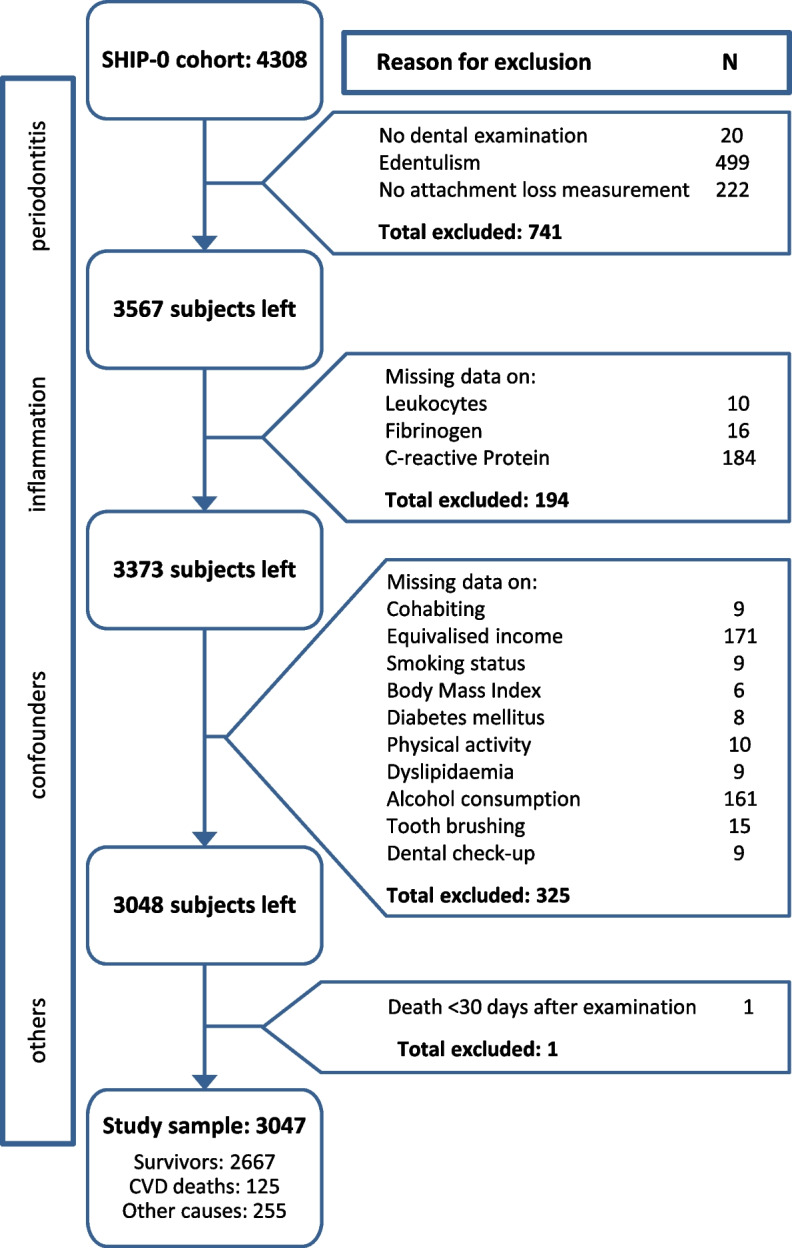
Exclusion criteria and missing data. Abbreviations: CVD, cardiovascular disease

Of the 4308 SHIP-START-0 participants, a total of 1261 subjects were excluded because of edentulism (*n* = 499), missing information on periodontitis (*n* = 242), inflammation (*n* = 194) or confounding variables (*n* = 325) and one due to death 5 days after SHIP-START-0 (Fig. 1). The final study population comprised 3047 subjects.

### Follow-up of vital status

Information on vital status was collected at varying intervals for up to 16.8 years from enrolment until March 2016, independent of further SHIP examination dates. Subjects were censored at death or loss to follow-up (e.g. migration). Mean follow-up was 13.0 years. For deceased subjects, death certificates were retrieved from local health authorities and coded according to the International Classification of Diseases, 10th revision (ICD10), by a board-certified nosologist. In addition, two internists independently validated the underlying causes of death and, in cases of disagreement, a third internist was consulted for a joint reading. CVD was considered the underlying cause of death for ICD10 codes I10 to I79.

### Dental examinations

The dental recording protocols for SHIP-START-0, SHIP-START-1 and SHIP-START-2 were identical [20]. Missing teeth as well as periodontal variables were determined excluding third molars. Pocket probing depth (PPD) and clinical attachment level (CAL) were assessed according to the half-mouth method at four sites per tooth (distobuccal, mesiobuccal, midbuccal, midlingual/midpalatal). PPD represents the distance from the gingival margin to the base of the periodontal pocket, while CAL represents the distance from the cementoenamel junction to the pocket base. CAL was not recorded if the cementoenamel junction was indistinct (e.g. wedge-shaped defects, fillings or crown margins).

For statistical analyses, mean values of PPD and CAL were calculated at the subject level. In addition, extent values were derived representing the percentage of sites with PPD/CAL ≥ 3/4 mm [21]. As tooth loss represents a consequence of advanced periodontitis [22], a combined periodontitis score was calculated as the mean of the z-transformed number of missing teeth, mean CAL and extent CAL ≥ 3 mm to represent lifetime accumulated periodontal impairment in one single metric variable.

All dental examinations were performed by trained and calibrated dentists. Calibration exercises were repeated every 6–12 months during data collection on volunteers not associated with the study. For CAL measurements, intraclass correlations per examiner ranged from 0.82 to 0.91 in SHIP-START-0 (SHIP-START-1: 0.70–0.89, SHIP-START-2: 0.76–0.88), whereas interclass correlations of 0.84, 0.90 and 0.74 were achieved in SHIP-START-0, SHIP-START-1 and SHIP-START-2, respectively.

### Laboratory measurements

At all SHIP examinations, non-fasting blood samples were taken from the cubital vein in the supine position. The samples were stored in the Integrated Research Biobank (IRB) of the University Medicine Greifswald and were used in accordance with the IRB regulations [23]. Leukocytes were determined by impedance measurement and fluorescence flow cytometry (SHIP-START-0/1: Coulter MaxM, Coulter Electronics, Miami, Florida; SHIP-START-2: XT 2000/XE 5000, Sysmex Corporation, Kobe, Japan). Fibrinogen concentrations were assayed according to Clauss (SHIP-START-0: Electra 1600, Instrumentation Laboratory, Barcelona, Spain; SHIP-START-1: Amax, Trinity Biotech, Bray, Ireland; SHIP-START-2: BCS-XP, Siemens Healthcare Diagnostics, Eschborn, Germany). C-reactive protein (CRP) levels were measured by particle-enhanced immunonephelometry (SHIP-START-0/1: Nephelometer II, Dade Behring Inc., Eschborn, Germany; SHIP-START-2: Dimension RxL/VISTA, Siemens Healthcare Diagnostics, Eschborn, Germany). As for periodontitis, a combined inflammation score was calculated as the mean of the *z*-transformed values for leukocytes, fibrinogen and CRP [24, 25].

In addition, total cholesterol, low-density lipoprotein cholesterol (LDL-C), high-density lipoprotein cholesterol (HDL-C) and serum glucose levels were determined (SHIP-START-0 1: Hitachi 704/717; Roche, Mannheim, Germany; SHIP-START-2: Dimension RxL/VISTA, Siemens Healthcare Diagnostics, Eschborn, Germany). Glycated haemoglobin A1c (HbA1c) was measured by high-performance liquid chromatography (SHIP-START-0/1: HPLC ClinRep, RECIPE Chemicals, Munich, Germany; SHIP-START-2: Diamat, Bio-Rad Laboratories, Hercules, California).

### Covariates

Sociodemographic, behavioural and medical covariates were consistently assessed in SHIP-START 0/1/2 using questionnaires, computer-assisted personal interviews and medical examinations. Sociodemographic information included age, sex, living with a partner (yes/no) and tertiles of equivalised household income (net household income divided by the square root of household size [26]). Behavioural risk factors covered smoking (never/former/current), physical activity, alcohol consumption and dental health behaviour. Participants were considered physically active if they reported at least 1 h of regular physical exercise per week in summer or winter. Alcohol consumption was assessed using a beverage-specific measure that multiplies standard ethanol content by drinking frequency and quantity [27]. High alcohol consumption was defined as an average daily intake of pure ethanol ≥ 30 g for men and ≥ 20 g for women. Dental health behaviour included regular professional supervision, defined as at least one dental check-up in the past year, and personal oral hygiene, defined as daily tooth brushing frequency ≥ 2. Obesity-related risk factors were also included. Participants were classified as normal weight (body mass index (BMI) < 25 kg/m^2^), overweight (BMI 25 to < 30 kg/m^2^) or obese (BMI ≥ 30 kg/m^2^). Diabetes mellitus [28], was assumed in case of self-reported physician’s diagnosis, antidiabetic treatment (Anatomical Therapeutic Chemical Classification System (ATC) code A10), or non-fasting glucose levels ≥ 11.1 mmol/l or HbA1c concentrations ≥ 48 mmol/mol (≥ 6.5%). Dyslipidaemia was defined as total cholesterol ≥ 6.2 mmol/l, LDL-C ≥ 4.1 mmol/l, HDL-C < 1.04 mmol/l, or the use of lipid-modifying agents (ATC code C10).

### Statistical analyses

Descriptive statistics included the calculation of means and standard deviations (SDs) for continuous variables (observation period, age, mean PPD, percentage of sites with PPD ≥ 3 mm, percentage of sites with PPD ≥ 4 mm, mean CAL, percentage of sites with CAL ≥ 3 mm, percentage of sites with CAL ≥ 4 mm, number of missing teeth, periodontitis score, leukocytes, fibrinogen, CRP and inflammation score) as well as the assessment of frequency distributions for categorical variables (sex, living in a partnership, equivalised income, smoking status, BMI, diabetes mellitus, physical activity, dyslipidaemia, high alcohol consumption, dental check-up during the last year and daily tooth brushing frequency ≥ 2). Depending on the scale, baseline differences between survivors and non-survivors and also between CVD deaths and non-CVD deaths were evaluated using *t*-tests or Mann–Whitney *U* tests (continuous) and *χ*^2^-tests (categorical). Changes in characteristics over time were then assessed using paired *t*-tests (continuous), McNemar’s tests (dichotomous) and Wilcoxon signed-rank tests (ordinal). The statistical methods for each of these comparisons are identified by letter codes in the respective tables and captions.

The probability of overall survival of study participants in terms of all-cause mortality, CVD mortality and non-CVD mortality was assessed graphically using Kaplan–Meier curves. For each measure of periodontitis and systemic inflammation, cut-off values were chosen to approximately divide the study population into one upper third (high category) and two lower thirds (low category, Additional file 1: Table S3 [29]). Considering all possible pairings of periodontitis and systemic inflammation measures, crude incidence rate ratios (IRR) and stratified Kaplan–Meier curves were determined for each scenario. Corresponding pairwise comparisons between the groups (healthy/high inflammation/high periodontitis/high inflammation and high periodontitis) using log-rank tests were corrected for multiple testing according to the Benjamini–Hochberg procedure.

Directed acyclic graphs (DAGs) were used to minimise bias in confounder selection [30, 31], as they perform equally well or better than conventional selection methods [32]. Using DAGitty [33], we constructed a number of different DAGs in which either inflammation, periodontitis or both factors were considered as exposures. The final DAG for the assessment of the interaction between systemic inflammation and periodontitis with respect to mortality is shown in Additional file 1: Figure S1. Accordingly, interaction models were adjusted for age, sex, living in a partnership, income, smoking status, BMI, diabetes mellitus, physical activity, dyslipidaemia, alcohol consumption, tooth brushing frequency and regular dental check-ups. The respective subsets for single exposure analyses are given in Additional file 1: Table S2.

Hazard ratios (HRs) and 95% confidence intervals (CIs) were obtained from multivariable Cox proportional hazards models to assess the association between measures of inflammation/periodontitis and all-cause, CVD and non-CVD mortality. Model assumptions were verified visually (hazard functions) and by means of *χ*^2^ tests based on Schoenfeld’s residuals. Although the proportional hazards assumption was met in the majority of tests, significant changes in study characteristics over time (Additional file 1: Table S1) required consideration of time-varying exposures and covariates via subject and variable-specific interval records [34]. In addition, two different Cox proportional hazards models are presented: one showing HRs per unit increase in each exposure variable to facilitate interpretation and one showing HRs per SD increase to allow comparison of effect sizes. Possible multiplicative and additive interactions between inflammation and periodontitis measures on mortality were analysed in joint Cox proportional hazards models. On the multiplicative scale, HRs were reported, and, on the additive scale, relative excess risks due to interaction (RERI) were calculated [35, 36]. Rothman refers to RERI as a biological interaction because its definition is based on the absence of biological independence of risks: the number of cases attributable to two risk factors is greater than the sum of cases caused by each factor alone. Analogously, an interaction on the multiplicative scale is referred to as a statistical interaction (combined risk > product of individual risks; statistical independence would imply equality) [36]. Since mediation and interaction are not mutually exclusive [37, 38], additional mediation models were constructed using the Stata module PARAMED [39] to assess the total, direct and indirect effects of inflammation on mortality via periodontitis and vice versa. Accordingly, relative risks (RRs) were reported for both mediation scenarios.

Several sensitivity analyses were performed considering different covariate definitions, e.g. for socioeconomic variables, smoking and diabetes, or excluding participants receiving cancer treatment, anti-inflammatory or immunosuppressive medication. In addition, models without the use of time-varying exposures and covariates, and thus potentially increased bias, were implemented. As cause-specific hazards may lead to inappropriate estimation of the cumulative incidence function, additional competing risk models [40] providing subdistribution hazards (SHR) were fitted for CDV mortality. However, there is no straightforward interpretation of the SHR [41, 42]. Therefore, this study focuses on cause-specific hazards as recommended when aetiology rather than prediction is of primary interest [41].

A posteriori power analyses were performed according to the formulae published by Peterson and George [43] and Van der Weele [44]. The calculations resulted in power measures between 0.86 and ≥ 0.99 for the study of individual effects and additive or multiplicative interactions on all-cause and CVD mortality with alpha = 0.05 and *n* = 3047. Regarding the interaction terms, two-sided *P* values < 0.10 were considered statistically significant. Otherwise, 0.05 was set as the cut-off for significance. The statistical analyses were performed using Stata/SE 14.2 [45] and cross-checked with R version 3.5 [46]. Some code examples in short Stata notation are given in Additional file 1: Table S6.

## Results

### Population characteristics and trends

A total of 3047 participants were monitored for a mean of 13.0 ± 2.4 years. During this period, 380 (12.5%) died from CVD (*n* = 125) or other causes (*n* = 255). Overall survival in terms of all-cause, CVD and non-CVD mortality is shown in Additional file 1: Figure S2. At baseline (Table 1), survivors were significantly younger, less likely to be male, had better periodontal and inflammatory parameters and lower frequencies of almost all risk factors considered. Interestingly, there were no significant differences in income and alcohol consumption, and the proportion of current smokers was higher among survivors (33.2%) than among non-survivors (23.7%). Regarding the cause of death, those who died from CVD were about 5 years older at baseline, more likely to have diabetes mellitus, less likely to brush their teeth and had about 2 fewer teeth than those who died from other causes (Table 1).

**Table 1:** Baseline characteristics of the entire study sample and stratified by survival and cause-specific mortality

	**Total**	**Survivors**	**All-cause deaths**	***P*-value**	**CVD deaths**	**Non-CVD deaths**	***P*-value**
*N*	3047	2667	380		125	255	
Observation period, years	13.0 ± 2.4	13.5 ± 1.2	9.0 ± 4.2	< 0.001^a^	8.8 ± 3.7	9.1 ± 4.5	0.412^b^
Sex (male)	1486 (48.8)	1232 (46.2)	254 (66.8)	< 0.001^c^	86 (68.8)	168 (65.9)	0.570^c^
Age, years	46.6 ± 15.2	44.1 ± 14.0	63.8 ± 12.0	< 0.001^a^	67.3 ± 10.3	62.2 ± 12.4	< 0.001^b^
Living in a partnership, yes	2348 (77.1)	2081 (78.0)	267 (70.3)	0.001^c^	83 (66.4)	184 (72.2)	0.249^c^
Equivalised income							
1st tertile, < 750 €	1019 (33.4)	903 (33.9)	116 (30.5)		35 (28.0)	81 (31.8)	
2nd tertile, 750–1200 €	1170 (38.4)	1004 (37.7)	166 (43.7)		65 (52.0)	101 (39.6)	
3rd tertile, > 1200 €	858 (28.2)	760 (28.5)	98 (25.8)	0.077^c^	25 (20.0)	73 (28.6)	0.056^c^
Smoking status							
Never smoker	1081 (35.5)	962 (36.1)	119 (31.3)		44 (35.2)	75 (29.4)	
Former smoker	990 (32.5)	819 (30.7)	171 (45.0)		58 (46.4)	113 (44.3)	
Current smoker	976 (32.0)	886 (33.2)	90 (23.7)	< 0.001^c^	23 (18.4)	67 (26.3)	0.204^c^
Body mass index							
< 25 kg/m^2^	1115 (36.6)	1039 (39.0)	76 (20.0)		19 (15.2)	57 (22.4)	
25– < 30 kg/m^2^	1208 (39.7)	1036 (38.9)	172 (45.3)		48 (38.4)	124 (48.6)	
≥ 30 kg/m^2^	742 (23.8)	592 (22.2)	132 (34.7)	< 0.001^c^	58 (46.4)	74 (29.0)	0.003^c^
Diabetes mellitus, yes	250 (8.2)	150 (5.6)	100 (26.3)	< 0.001^c^	37 (29.6)	63 (24.7)	0.309^c^
Physical activity, yes	1406 (46.1)	1283 (48.1)	123 (32.4)	< 0.001^c^	34 (27.2)	89 (34.9)	0.132^c^
Dyslipidaemia, yes	1415 (46.4)	1170 (43.9)	245 (64.5)	< 0.001^c^	84 (67.2)	161 (63.1)	0.437^c^
High alcohol consumption, yes	446 (14.6)	393 (14.7)	53 (14.0)	0.684^c^	19 (15.2)	34 (13.3)	0.622^c^
Dental check-up during the last year, yes	2729 (89.6)	2408 (90.3)	321 (84.5)	0.001^c^	104 (83.2)	217 (85.1)	0.631^c^
Daily tooth brushing frequency ≥ 2, yes	2531 (83.1)	2268 (85.0)	263 (69.2)	< 0.001^c^	76 (60.8)	187 (73.3)	0.013^c^
Mean PPD, mm	2.5 ± 0.7	2.4 ± 0.7	2.9 ± 0.9	< 0.001^a^	2.9 ± 0.9	2.9 ± 0.9	0.826^a^
Percentage of sites with PPD ≥ 3 mm, %	44.9 ± 24.2	43.1 ± 23.5	57.7 ± 24.7	< 0.001^a^	57.7 ± 25.1	57.7 ± 24.5	0.994^a^
Percentage of sites with PPD ≥ 4 mm, %	12.3 ± 16.9	11.0 ± 15.6	21.0 ± 22.2	< 0.001^b^	21.1 ± 22.5	21.0 ± 22.1	0.970^b^
Mean CAL, mm	2.6 ± 1.9	2.3 ± 1.7	4.4 ± 2.1	< 0.001^a^	4.7 ± 2.3	4.2 ± 1.9	0.115^b^
Percentage of sites with CAL ≥ 3 mm, %	46.0 ± 34.8	41.4 ± 33.5	77.8 ± 26.0	< 0.001^b^	80.8 ± 23.3	76.6 ± 27.2	0.272^b^
Percentage of sites with CAL ≥ 4 mm, %	27.4 ± 31.7	22.9 ± 28.8	58.9 ± 32.8	< 0.001^b^	62.1 ± 32.1	57.3 ± 33.1	0.153^b^
Number of missing teeth	7.1 ± 6.8	6.2 ± 6.2	13.6 ± 7.7	< 0.001^b^	15.0 ± 7.0	12.8 ± 7.8	0.010^b^
Periodontitis score	0.0 ± 0.9	− 0.1 ± 0.8	0.9 ± 0.8	< 0.001^a^	1.1 ± 0.7	0.8 ± 0.8	0.012^a^
Leukocytes, Gpt/l	6.7 ± 2.0	6.7 ± 1.9	7.1 ± 2.5	< 0.001^a^	7.1 ± 2.3	7.1 ± 2.6	0.967^a^
Fibrinogen, g/l	2.9 ± 0.7	2.9 ± 0.7	3.2 ± 0.8	< 0.001^a^	3.3 ± 0.8	3.2 ± 0.8	0.567^a^
C-reactive protein, mg/l	2.7 ± 5.4	2.4 ± 4.3	4.3 ± 10.1	< 0.001^b^	5.8 ± 16.2	3.6 ± 4.6	0.360^b^
Inflammation score	0.0 ± 0.8	− 0.1 ± 0.7	0.4 ± 0.9	< 0.001^a^	0.4 ± 0.9	0.3 ± 0.8	0.454^a^

Data are presented as mean ± standard deviation or number (percentage)

Equivalised income: net household income divided by the square root of household size. Diabetes mellitus: self-reported physician’s diagnosis or antidiabetic treatment or non-fasting glucose levels ≥ 11.1 mmol/l or glycated haemoglobin concentrations ≥ 48 mmol/mol. Physical activity: at least 1 h of physical exercise per week during summer or winter. Dyslipidaemia: total cholesterol ≥ 6.2 mmol/l, low density lipoprotein cholesterol ≥ 4.1 mmol/l, high density lipoprotein cholesterol < 1.04 mmol/l or use of lipid-modifying agents. High alcohol consumption: average pure ethanol intake of ≥ 30 g per day for men and ≥ 20 g per day for women. Periodontitis score: mean of *z*-transformed values for missing teeth, mean CAL and percentage of sites with CAL ≥ 3 mm. Inflammation score: mean of *z*-transformed values for leukocytes, fibrinogen and C-reactive protein

*Abbreviations*: *CVD* cardiovascular disease, *PPD* pocket probing depth, *CAL* clinical attachment level

*P*-values were obtained using *t*-tests (continuous, normally distributed variables: a), Mann–Whitney *U* tests (continuous, not normally distributed variables: b) and *χ*^2^-tests (categorical variables: c)

Changes in sample characteristics over the course of the study are shown in Additional file 1: Table S1. From SHIP-START-0 to SHIP-START-2, most of the assessed factors underwent significant changes. When the analysis was restricted to the 1852 participants who survived to SHIP-START-2 and attended the second follow-up, the proportion of current smokers (SHIP-START-0: 28.8%; SHIP-START-2: 20.7%; *P* < 0.001) and subjects reporting high alcohol consumption (SHIP-START-0: 15.4%; SHIP-START-2: 10.3%; *P* < 0.001) decreased. Although more subjects reported regular physical activity (SHIP-START-0: 49.1%; SHIP-START-2: 68.1%; *P* < 0.001), the proportion of obese subjects (SHIP-START-0: 21.8%; SHIP-START-2: 32.5%; *P* < 0.001) and those with diabetes mellitus (SHIP-START-0: 5.4%; SHIP-START-2: 12.7%; *P* < 0.001) increased significantly. Furthermore, all periodontal variables showed progression (e.g. for mean CAL: SHIP-START-0: 2.4 ± 1.5 mm; SHIP-START-2: 2.9 ± 1.7 mm; *P* < 0.001), whereas oral health behaviour did not change substantially over time (Additional file 1: Table S1).

### Inflammation and periodontitis as risk factors for mortality

**Fig. 2:**
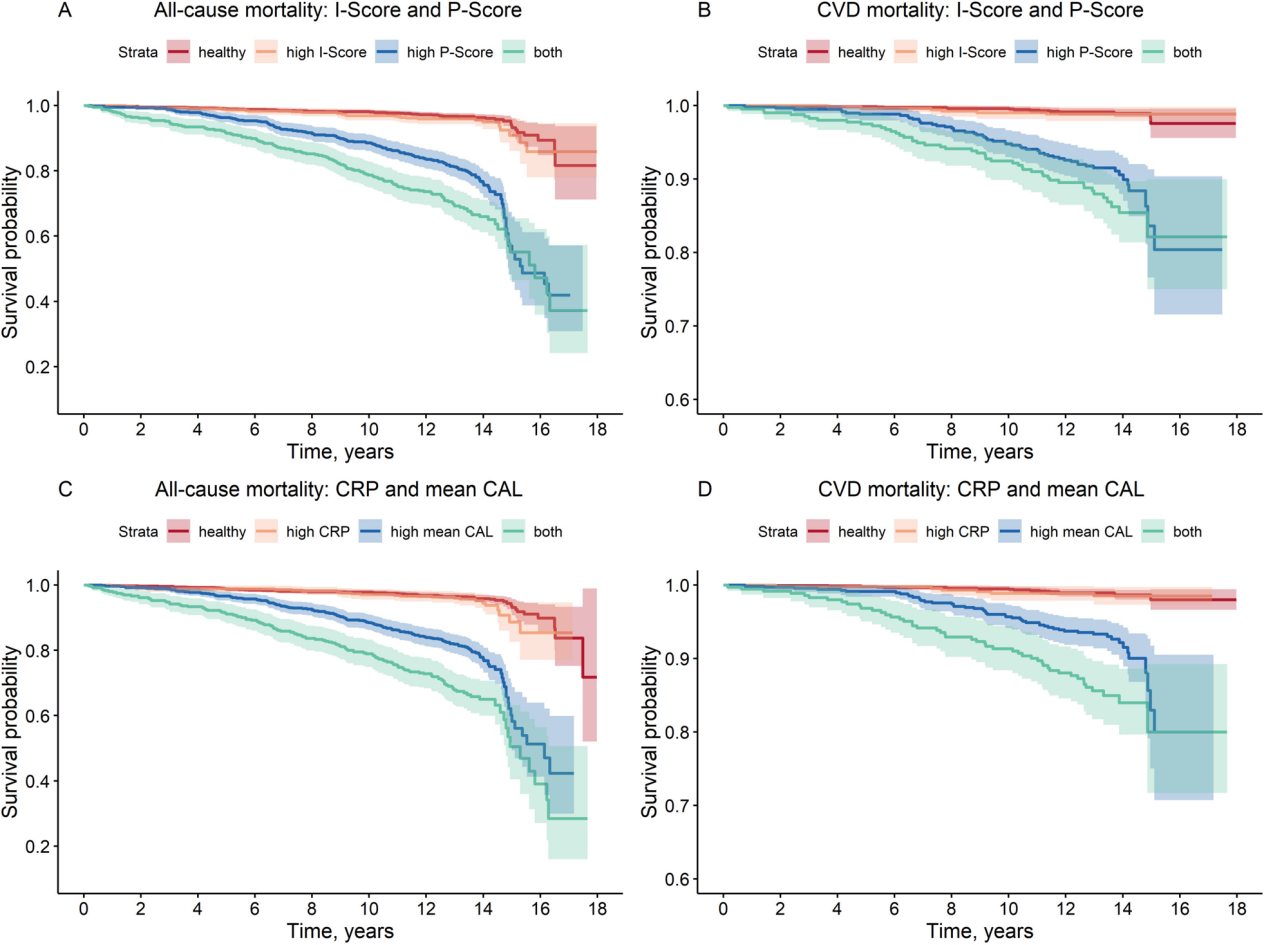
Survival probability stratified by I-Score, P-Score, CRP and mean CAL. Survival probabilities of study participants regarding all-cause mortality according to inflammation score and periodontitis score (**A**), CVD mortality according to inflammation score and periodontitis score (**B**), all-cause mortality according to CRP and mean CAL (**C**) and CVD mortality according to CRP and mean CAL (**D**). Detailed information on subjects at risk and numbers of events is given in Additional file 1: Figure S3. Cut-off values for strata definition: I-Score: 0.3; P-Score: 0.3; CRP: 2.7 mg/l; mean CAL: 3.0 mm. Abbreviations: I-Score, inflammation score; P-Score, periodontitis score; CVD, cardiovascular disease; CRP, C-reactive protein; CAL, clinical attachment level

Survival of study participants with respect to all-cause and CVD mortality in relation to inflammation and periodontitis is shown in Fig. 2 and in Additional file 1: Figure S3, which additionally provides detailed information on corresponding events and person-years. It can be seen that there were no major differences in the stratified curve shapes according to cause of death (Fig. 2A vs. B or C vs. D). The same applies to the choice of variables to define low/high categories of inflammation and periodontitis (Fig. 2A vs. C or B vs. D). With this in mind, Additional file 1: Figure S4 shows additional survival curves stratified by further periodontitis/inflammation combinations. Overall, no significant differences were observed between the healthy participants and those with only high levels of inflammation. In comparison, participants with high levels of periodontal variables had significantly lower survival than the two groups mentioned above, while the lowest survival was observed in the group with both high inflammation and high periodontitis measures (Fig. 2, Figs. S2 and S3).

Using Cox proportional hazards models with full adjustment for time-varying exposures and covariates, all inflammation markers demonstrated apparent associations with all-cause mortality (HR per SD increase: leukocytes: 1.264 (95% CI: 1.163–1.374), fibrinogen: 1.120 (95% CI: 1.030–1.218), CRP: 1.231 (95% CI: 1.109–1.366), inflammation score: 1.358 (95% CI: 1.210–1.523), Table 2). Quite similar results in terms of effect size and confidence intervals were found for CVD and non-CVD mortality (Table 2). However, compared with the results for CAL and missing teeth, the PPD measures showed only weak associations with mortality, as indicated by smaller effect sizes. For CVD mortality, neither mean PPD nor extent PPD ≥ 3 mm/4 mm showed a relevant association (Table 2). Classical Cox proportional hazards models with fixed baseline exposures and covariates (Additional file 1: Table S4) confirmed these results.

**Table 2:** Effects of periodontitis and systemic inflammation measures on all-cause, CVD and non-CVD mortality

Measure	All-cause mortality			CVD mortality			Non-CVD mortality		
	HR (95% CI)		*P*-value	HR (95% CI)		*P*-value	HR (95% CI)		*P*-value
	Per 1-unit increase	Per SD increase		Per 1-unit increase	Per SD increase		Per 1-unit increase	Per SD increase	
**Periodontitis**^**a**^									
Mean PPD, mm	1.097 (0.983; 1.224)	1.068 (0.988; 1.155)	0.098	1.111 (0.920; 1.341)	1.078 (0.942; 1.233)	0.275	1.100 (0.961; 1.258)	1.070 (0.972; 1.178)	0.166
Sites with PPD ≥ 3 mm, %	1.005 (1.000; 1.009)	1.125 (1.015; 1.247)	0.025	1.004 (0.997; 1.011)	1.096 (0.921; 1.305)	0.302	1.006 (1.001; 1.011)	1.151 (1.014; 1.308)	0.030
Sites with PPD ≥ 4 mm, %	1.005 (1.000; 1.010)	1.091 (1.008; 1.181)	0.030	1.004 (0.996; 1.012)	1.070 (0.932; 1.228)	0.337	1.006 (1.000; 1.012)	1.108 (1.006; 1.220)	0.037
Mean CAL, mm	1.104 (1.051; 1.161)	1.205 (1.097; 1.323)	< 0.001	1.114 (1.022; 1.214)	1.225 (1.042; 1.439)	0.014	1.101 (1.036; 1.171)	1.199 (1.069; 1.346)	0.002
Sites with CAL ≥ 3 mm, %	1.010 (1.006; 1.015)	1.429 (1.223; 1.670)	< 0.001	1.007 (0.999; 1.015)	1.288 (0.976; 1.702)	0.074	1.012 (1.006; 1.017)	1.504 (1.246; 1.815)	< 0.001
Sites with CAL ≥ 4 mm, %	1.008 (1.005; 1.012)	1.292 (1.156; 1.444)	< 0.001	1.006 (1.000; 1.012)	1.217 (1.002; 1.479)	0.047	1.009 (1.005; 1.013)	1.334 (1.164; 1.528)	< 0.001
Number of missing teeth	1.023 (1.009; 1.038)	1.180 (1.065; 1.307)	0.002	1.029 (1.003; 1.055)	1.226 (1.025; 1.466)	0.026	1.021 (1.003; 1.039)	1.158 (1.021; 1.313)	0.022
Periodontitis score	-	1.394 (1.202; 1.616)	< 0.001	-	1.399 (1.076; 1.820)	0.012	-	1.399 (1.169; 1.676)	< 0.001
**Systemic inflammation**^**b**^									
Leukocytes, Gpt/l	1.128 (1.081; 1.178)	1.264 (1.163; 1.374)	< 0.001	1.152 (1.071; 1.238)	1.316 (1.143; 1.514)	< 0.001	1.118 (1.060; 1.180)	1.242 (1.119; 1.378)	< 0.001
Fibrinogen, g/l	1.163 (1.040; 1.300)	1.120 (1.030; 1.218)	0.008	1.174 (0.975; 1.414)	1.128 (0.981; 1.297)	0.090	1.160 (1.009; 1.335)	1.118 (1.007; 1.242)	0.037
CRP, mg/l, log-transformed	1.204 (1.097; 1.322)	1.231 (1.109; 1.366)	< 0.001	1.209 (1.025; 1.427)	1.236 (1.028; 1.488)	0.024	1.201 (1.073; 1.345)	1.227 (1.081; 1.392)	0.002
Inflammation score	-	1.358 (1.210; 1.523)	< 0.001	-	1.385 (1.141; 1.681)	0.001	-	1.351 (1.172; 1.558)	< 0.001

Results from Cox-proportional hazards models with consideration of time-varying exposures and covariates via subject and variable-specific interval records. A separate model was calculated for each exposure/mortality combination. Corresponding effect estimates from models with classical time-invariant exposures and adjustments are given in Additional file 1: Table S4

*Abbreviations*: *CVD* cardiovascular disease, *PPD* pocket probing depth, *CAL* clinical attachment level, *CRP* C-reactive protein, *HR* hazard ratio, *CI* confidence interval, *SD* standard deviation

^a^Models were adjusted for time-varying values of age, sex, living in a partnership, tertiles of equivalised income, smoking status, categories of body mass index, diabetes mellitus, physical activity, tooth brushing frequency and regular dental check-ups

^b^Models were adjusted for time-varying values of age, gender, living in a partnership, tertiles of equivalised income, smoking status, categories of body mass index, diabetes mellitus, physical activity, dyslipidaemia and high alcohol consumption

### Interaction between periodontitis and systemic inflammation

**Fig. 3:**
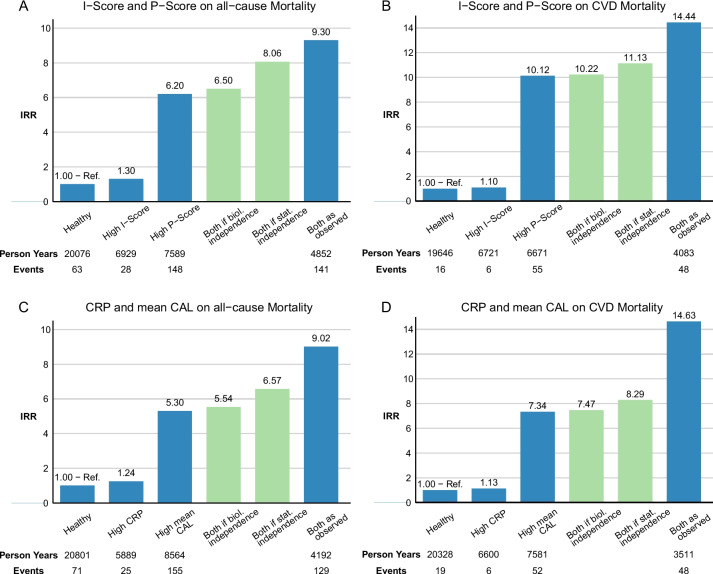
Incidence rate ratios for the interaction of systemic inflammation and periodontitis on mortality. Observed crude incidence rate ratios for the interaction between inflammation score and periodontitis score regarding all-cause mortality (**A**) and CVD mortality (**B**) as well as the interaction between C-reactive protein and mean clinical attachment level regarding all-cause mortality (**C**) and CVD mortality (**D**). Blue bars represent observed IRRs whereas green bars represent the expected IRRs for the strata having high measures for both, inflammation and periodontitis, in case of biological (additive scale) or statistical (multiplicative scale) independence of effects. Cut-off values for strata definition: I-Score: 0.3; P-Score: 0.3; CRP: 2.7 mg/l; mean CAL: 3.0 mm. Abbreviations: IRR, incidence rate ratio; I-Score, inflammation score; P-Score, periodontitis score; CVD, cardiovascular disease; CRP, C-reactive protein; CAL, clinical attachment level

Figure 3 and Additional file 1: Figure S5 visualise the observed IRRs together with the IRRs that would be expected if inflammation and periodontitis were biologically or statistically independent. As for the survival curves, neither the type of mortality (Fig. 3A vs. B or C vs. D) nor the categorising variables for periodontitis and inflammation (Fig. 3A vs. C or B vs. D; Additional file 1: Figure S5) changed the overall proportions. Participants with high inflammation showed slightly increased IRRs compared to the healthy reference, whereas a considerable increase was observed in the high periodontitis group.

Compared with the theoretically expected IRRs, the observed values for participants with high inflammation and periodontitis were always significantly greater than those representing biological independence and greater to approximately equal to the expected IRRs in the case of statistical independence. This was also true when the study sample was restricted to certain confounder levels, such as males, those without diabetes mellitus or never smokers; the IRRs of the last-mentioned group are shown in Additional file 1: Figure S6 with respect to all-cause mortality.

Nevertheless, extensive modelling of the interactions revealed some variation with respect to the type of mortality and the choice of exposition variables. For the combination of inflammation score and periodontitis score, notable additive interactions were observed for all-cause and CVD mortality, indicating an excess mortality risk of approximately 18.9% and 27.8%, respectively (Table 3). For CRP and mean CAL, there was a 14.8% excess risk for CVD mortality (Table 3). Overall, significant additive interaction terms were observed for all-cause and especially CVD mortality but not in analyses of non-CVD mortality (except for the combination of leukocytes and extent CAL, Additional file 1: Table S5). Similarly, the RERIs for CVD mortality tended to be larger than those for all-cause mortality (Table 3, Additional file 1: Table S5). In terms of multiplicative interactions, the choice of periodontal variable appeared to be crucial, as consistent results only occurred when mean PPD or extent PPD ≥ 4 mm was considered together with fibrinogen, CRP or inflammation score (Additional file 1: Table S5). However, in line with the RERI analyses, the multiplicative interactions of PPD and inflammation markers were stronger for CVD mortality (HRs ranged from 1.12 to 1.18; extent PPD and fibrinogen: HR = 1.147, *P* = 0.006; extent PPD and inflammation score: HR = 1.134, *P* = 0.042) than for all-cause mortality (extent PPD and fibrinogen: HR = 1.075, *P* = 0.018; extent PPD and inflammation score: HR = 1.069, *P* = 0.073) and did not reach statistical significance for non-CVD mortality (Additional file 1: Table S5).

**Table 3:** Interaction and mediation of periodontitis and systemic inflammation regarding mortality

	All-cause mortality				CVD mortality				Non-CVD mortality			
	I-Score/P-Score		CRP/mean CAL		I-Score/P-Score		CRP/mean CAL		I-Score/P-Score		CRP/mean CAL	
	HR (95% CI)	*P*-value	HR (95% CI)	*P*-value	HR (95% CI)	*P*-value	HR (95% CI)	*P*-value	HR (95% CI)	*P*-value	HR (95% CI)	*P*-value
**Interaction**												
Model A: Fully adjusted cox proportional hazards model												
Inflammation measure	1.335 (1.188; 1.501)	< 0.001	1.191 (1.071; 1.325)	0.001	1.361 (1.113; 1.663)	0.003	1.172 (0.969; 1.417)	0.101	1.330 (1.151; 1.537)	< 0.001	1.198 (1.054; 1.363)	0.006
Periodontitis measure	1.380 (1.191; 1.599)	< 0.001	1.184 (1.079; 1.300)	< 0.001	1.384 (1.063; 1.801)	0.016	1.204 (1.023; 1.417)	0.025	1.380 (1.154; 1.649)	< 0.001	1.174 (1.047; 1.316)	0.006
Model B: Model A + inclusion of a multiplicative interaction term and post hoc RERI calculation												
Inflammation measure	1.268 (1.053; 1.528)	0.012	1.201 (1.050; 1.374)	0.007	1.174 (0.816; 1.689)	0.387	1.014 (0.781; 1.315)	0.919	1.328 (1.069; 1.650)	0.010	1.282 (1.099; 1.496)	0.002
Periodontitis measure	1.357 (1.164; 1.583)	< 0.001	1.190 (1.072; 1.320)	0.001	1.320 (1.000; 1.744)	0.050	1.120 (0.929; 1.351)	0.236	1.379 (1.145; 1.661)	0.001	1.222 (1.079; 1.385)	0.002
Multiplicative interaction	1.054 (0.910; 1.220)	0.482	0.991 (0.910; 1.079)	0.838	1.143 (0.872; 1.499)	0.333	1.129 (0.976; 1.306)	0.102	1.001 (0.840; 1.194)	0.987	0.922 (0.830; 1.025)	0.132
	RERI (95% CI)	*P*-value	RERI (95% CI)	*P*-value	RERI (95% CI)	*P*-value	RERI (95% CI)	*P*-value	RERI (95% CI)	*P*-value	RERI (95% CI)	*P*-value
Additive Interaction	0.189 (0.000; 0.378)	0.050	0.026 (− 0.072; 0.123)	0.607	0.278 (− 0.025; 0.581)	0.072	0.148 (0.013; 0.283)	0.032	0.127 (− 0.116; 0.370)	0.305	− 0.059 (− 0.190; 0.072)	0.378
**Mediation**												
Model C: Mediation analysis considering inflammation as a mediator for the association between periodontitis and mortality												
Total effect	1.322 (1.121; 1.559)	0.001	1.143 (1.077; 1.214)	< 0.001	1.304 (0.968; 1.757)	0.080	1.020 (0.898; 1.160)	0.756	1.338 (1.096; 1.633)	0.004	1.176 (1.096; 1.262)	< 0.001
Direct effect	1.304 (1.106; 1.538)	0.002	1.134 (1.069; 1.202)	< 0.001	1.285 (0.955; 1.730)	0.098	1.028 (0.910; 1.161)	0.656	1.321 (1.082; 1.613)	0.006	1.160 (1.082; 1.243)	< 0.001
Indirect effect	1.014 (1.002; 1.026)	0.027	1.008 (1.001; 1.016)	0.027	1.015 (0.995; 1.035)	0.140	0.993 (0.979; 1.006)	0.291	1.013 (0.998; 1.027)	0.080	1.014 (1.005; 1.024)	0.002
Model D: Mediation analysis considering periodontitis as a mediator for the association between inflammation and mortality												
Total effect	1.187 (0.982; 1.436)	0.076	1.108 (0.982; 1.250)	0.096	1.089 (0.742; 1.597)	0.664	0.942 (0.743; 1.193)	0.618	1.226 (0.985; 1.525)	0.068	1.175 (1.022; 1.351)	0.024
Direct effect	1.166 (0.956; 1.421)	0.046	1.101 (0.975; 1.244)	0.121	1.065 (0.714; 1.589)	0.756	0.934 (0.736; 1.186)	0.574	1.204 (0.959; 1.512)	0.110	1.169 (1.015; 1.346)	0.030
Indirect effect	1.019 (1.000; 1.037)	0.095	1.006 (1.000; 1.012)	0.041	1.022 (0.990; 1.055)	0.182	1.008 (0.998; 1.018)	0.099	1.018 (0.997; 1.040)	0.098	1.005 (0.998; 1.012)	0.177

Results are given per 1 SD increase. Cox proportional hazards models were adjusted for time-varying values of age, sex, living in a partnership, tertiles of equivalised income, smoking status, categories of body mass index, diabetes mellitus, physical activity, dyslipidaemia, high alcohol consumption, tooth brushing frequency and regular dental check-ups. In each case, one inflammation measure and one periodontitis measure were analysed conjointly

*Abbreviations*: *CVD* cardiovascular disease, *I-Score* inflammation score, *P-Score* periodontitis score, *CRP* C-reactive protein, *CAL* clinical attachment level, *HR* hazard ratio, *RR* relative risk, *RERI* relative excess risk due to interaction, *SD* standard deviation

### Mediation of effects

The purpose of the mediation analyses was to examine how the predictor variable (*X* = periodontitis in model C/inflammation in model D) influences the survival outcome (*Y* = all-cause mortality/CVD mortality/non-CVD mortality) through the mediator variable (*M* = inflammation in model C/periodontitis in model D). The direct effect of *X* on *Y* refers to the effect that is not explained by *M*, while the indirect effect of *X* on *Y* refers to the effect that is mediated by *M*. In the present mediation models (Table 3, lower part), the total effects of *X* on *Y* were roughly equal to the estimated direct effects, while most indirect effects were marginal (5–6% of the total effects). For example, in model C for all-cause mortality, the estimated RR for the direct effect of periodontitis score was 1.304, corresponding to a 30.4% increase in mortality risk per unit increase in periodontitis score not explained by the inflammation score. Similarly, the RR of 1.014 for the indirect effect represents a 1.4% increase in mortality risk per unit increase in periodontitis score mediated by the inflammation score. Thus, the total effect of the periodontitis score on all-cause mortality (RR = 1.304*1.014 = 1.322, Table 3) is divided into approximately 95% direct effect and 5% indirect effect. The indirect effects in this study were not only very small but also inconsistent in direction and statistical significance (largest observed indirect effect: RR = 1.022, *P* = 0.182). This was true for both scenarios, with either inflammation (model C) or periodontitis (model D) as a potential mediator (Table 3, lower part). In summary, the mediation analyses did not reveal a clear indirect effect on mortality.

## Discussion

To our knowledge, this is the first population-based longitudinal study to evaluate the impact of periodontitis, systemic inflammation and their complex interactions on all-cause and CVD mortality. A 1-unit increase in the periodontitis score was associated with a 40% higher risk of mortality, regardless of the underlying cause of death, and a 1-unit increase in the inflammation score was associated with a 35% to 39% higher risk of mortality. On the additive scale, the interaction of periodontitis and systemic inflammation was associated with excess risks of 19% for all-cause mortality and 28% for CVD mortality. However, there was no evidence of an effect of periodontitis mediated through inflammation and vice versa.

With a global prevalence of 11.2% for severe periodontitis [17] and a number of systemic diseases associated with periodontitis such as diabetes, rheumatoid arthritis, CVD and others, periodontitis is presently considered a risk factor for general health [16, 47, 48]. However, findings on the association between periodontitis and mortality were inconclusive: while an association between periodontal disease and an increased risk of fatal CHD was observed in 10,368 participants in the Nutrition Canada Survey [49], and poor oral health was associated with all-cause mortality [50], many other studies reported conflicting results, most likely due to small sample sizes [51], self-reporting of periodontitis [52, 53] or aspects of statistical analysis [54]. In a recent publication of 506 participants from the Kuopio Oral Health and Heart Study who were monitored for 20 years, edentulism but not periodontitis increased the risk of CVD and all-cause mortality [55]. In large and longitudinal studies, tooth loss or the number of missing teeth is often used as an indicator of periodontitis because it is less susceptible to error in repeated measurements: In a Finnish population-based survey of 8446 subjects with 13 years of follow-up, it was observed that ≥ 5 missing teeth as a surrogate for baseline periodontitis were significantly associated with coronary heart disease (HR = 1.6) and acute myocardial infarction (HR = 2.4) whereas ≥ 9 missing teeth were associated with all-cause mortality (HR = 1.4) [56]. In particular, the measurement of periodontitis has proven to be an obstacle, and recent publications still point to the limited usefulness of improperly collected or self-reported periodontal data [57, 58]. Nevertheless, many well-established studies have now confirmed periodontitis as an independent risk factor for all-cause, CVD and other cause-specific mortality [59–61]. Most notably, a recent comprehensive review and meta-analyses of pooled data from 47 studies involved more than 5.71 million participants and a total of approximately 149,000 deaths. The reported risk ratios for the association of periodontitis with all-cause and CVD mortality were 1.46 (95% CI: 1.15–1.85; based on 19 studies, *n* ≈ 640.000) and 1.47 (95% CI: 1.14–1.90; based on 11 studies, *n* ≈ 376.000), respectively [62]. There are obvious limitations to meta-analyses, for example in terms of adjustment and cohort heterogeneity, but the effect sizes reported are essentially consistent with our findings.

The biological mechanisms linking periodontitis to systemic diseases, including CVD, are quite complex. Periodontitis contributes to systemic inflammation by promoting bacteraemia during personal oral hygiene, chewing or dental treatment [9] and also by the continuous leakage of locally produced inflammatory mediators from the impaired gingival tissue into the bloodstream [11, 12]. At the same time, these mediators are involved in all stages of atherosclerosis, from the initiation of lesions to the triggering of adverse events [2–4]. However, the interplay between periodontitis and systemic inflammation in terms of their impact on mortality risk is still unclear. The mutual influence of both conditions is widely accepted, and although the impact of periodontitis on inflammation markers [63] as well as the long-term impact of low-grade inflammation on periodontitis progression [64] have been reported in the SHIP cohort, little is known about their biological interaction or mediation of effects on CVD events or death. Two recent studies have attempted to address this issue using mediation analysis. Van Dyke et al. analysed data from 304 subjects using advanced imaging, namely ^18^F-fluorodeoxyglucose positron emission tomography/computed tomography (^18^F-FDG-PET/CT), to quantify both periodontal and arterial inflammation [65]. For the 13 major CVD events observed, periodontal inflammation emerged as a significant predictor with a 2.25-fold increased risk per SD increase, whereas periodontal bone loss did not. Subsequent mediation analyses showed that arterial inflammation accounted for approximately 80% of this observed effect [65]. These findings contrast with the marginal mediation observed in our data (5–6%), suggesting that ^18^F-FDG-PET/CT may capture another aspect of the relationship between periodontitis and systemic inflammation that is more directly related to CVD and less diluted by other effects. In addition, the comparability of results is generally limited by the highly selected study sample and the small number of events, including only one CVD death [65]. Another study analysed data from the Northern Ireland Prospective Epidemiological Study of Myocardial Infarction (PRIME). During 17 years of follow-up, 500 of 1558 men died and accelerated failure time modelling revealed a 15% reduction in survival relative to a doubling of CAL, with no more than 10% of this effect being mediated by CRP [66]. Overall, compared with men with no/mild periodontitis, those with severe periodontitis (using the Centers for Disease Control and Prevention/American Academy of Periodontitis (CDC/AAP) case definition) had an HR of 1.34 (95% CI: 1.06–1.70) for all-cause mortality [66]. Although this cohort was exclusively male and had a small age range (58–72 years), the results are in good agreement with our observations.

The absence of major effect mediation in SHIP and PRIME does not imply the absence of interdependencies between periodontitis and systemic inflammation with respect to CVD or mortality. For the vast majority of variable combinations, we observed substantially higher mortality rates in participants with both periodontitis and increased systemic inflammation than would be theoretically expected if the effects were biologically or statistically independent (Fig. 3, Additional file 1: Figure S5). Accordingly, we observed a clear interaction of the two exposures with an excess risk of up to 28% on the additive scale (RERI), indicating that the combined effect of periodontitis and systemic inflammation on mortality is up to 28% greater than the sum of their individual effects. In other words, the combined effect *E*(A = 1, *B* = 1) of high periodontitis score (here *A* = 1) and high inflammation score (here *B* = 1) can be calculated as *E*(*A* = 1, *B* = 1) = *E*(*A* = 1) + *E*(*B* = 1) + 0.28 × [*E*(*A* = 1) + *E*(*B* = 1)]. If we would have had instead observed a relevant multiplicative interaction of 28% between high periodontitis score and high inflammation score, the combined effect would have been calculated as *E*(*A* = 1, *B* = 1) = *E*(*A* = 1) + *E*(*B* = 1) + 0.28 × [*E*(*A* = 1) × *E*(*B* = 1)]. This constructed example shows one different symbol with great impact, depending on the size of *E*(*A* = 1) and *E*(*B* = 1). Thus, it is not surprising that interaction analysis on the multiplicative scale yielded different results than the additive scale. Although the multiplicative approach is the fastest and simplest and thereby most commonly used way to test for interaction, it is not generally suitable for investigating biological relationships by definition [36]. Furthermore, it is advisable to always report both scales when examining interaction [67]. Moreover, it should be noted that lack of interaction on either scale is a frequent occurrence. Even contrasting directions of interaction can occur on both scales and would not signify calculation errors [67]. While multiplicative interaction is easier to calculate, additive interaction is much more powerful in defining subpopulations that would benefit most from, for example, a therapy with limited availability. Therefore, it is strongly recommended to consider additive interaction, previously known as synergism, particularly in public health settings [36, 67, 68].

In the particular context of our investigation, the additional mortality risk of up to 28% due to the interaction of periodontitis and systemic inflammation suggests that this subpopulation would benefit most from both periodontal and anti-inflammatory therapies.

However, the observed variance in RERI with the choice of exposure combinations in additive interaction analyses provided an interesting picture of possible underlying biological mechanisms, as the different markers represent different aspects of the disease (Additional file 1: Table S5). Combinations of periodontitis score with inflammation score and mean CAL with CRP were the main focus, but Additional file 1: Table S5 provides a summary for all 96 different pairings (8 periodontal variables × 4 inflammation variables × 3 mortality types): there was a noticeable additive interaction between measures of PPD, which best reflects current periodontal inflammation, and fibrinogen, CRP and inflammation score, but not with leukocyte levels. At the same time, measures of CAL, which is considered a strong proxy for the lifetime bacterial and inflammatory burden of the periodontium, showed the strongest interactions with CRP, leukocytes and inflammation score, but not with fibrinogen. On the contrary, the number of missing teeth was the only dental variable that showed no apparent interaction with CRP, which is not surprising given that tooth loss, although robust and not prone to measurement error, provides limited information on the inflammatory burden as the reasons for tooth extraction are quite heterogeneous [22] and periodontitis accounts for at most 30% of extracted teeth [69]. With such a plethora of markers and their multiple interrelationships, it is very complex to compare the results of different studies, which is why we decided to use aggregated scores. But although the use of scores is not uncommon [24, 25, 50], the results still depend on the individual composition. However, in terms of current clinical practice, easily measurable means will always be essential.

To date, there is no profound consensus on whether and how periodontal treatment may have beneficial effects on atherosclerosis, CVD events and survival [70]. Nevertheless, recent studies suggest some potential. Both local and systemic inflammatory responses following periodontal therapy have been well documented [47]. In addition, periodontal therapy has also been associated with a consistent long-term reduction in inflammation as measured by CRP, IL-6 and fibrinogen [71] as well as significantly improved endothelial function up to 6 months post-treatment [72]. In contrast, in a randomised controlled trial of 90 patients with peripheral arterial disease and severe periodontitis, no reduction in vascular inflammation as determined by ^18^F-FDG-PET/CT was seen 3 months after periodontal therapy with or without additional systemic antibiotics [73]. However, in 3631 haemodialysis patients, intensive periodontal therapy was associated with an HR of 0.78 for CVD hospital admission and 0.49 for all-cause mortality compared with untreated controls [74]. In addition, a population-based observational study of 247,696 Koreans reported a 14% lower risk of CVD events in individuals with regular dental visits for professional cleaning [75]. Moreover, in a nationwide study in Taiwan, the incidence rate of acute myocardial infarction was 3.5% in 7164 participants who did not receive dental scaling, almost twice as high as in the matched control group (1.9%) who received regular scaling [76]. Finally, several systematic reviews agreed that despite evidence of beneficial effects of periodontal treatment on surrogate measures of CVD, there is a lack of adequate randomised controlled trials focusing on hard CVD endpoints to reasonably support or refute preventive effects [47, 70, 77].

Nevertheless, it is widely believed that reducing systemic inflammation will also reduce the risk of atherosclerosis and CVD events [5], and extensive research is underway to identify suitable agents. For example, the Canakinumab Anti-Inflammatory Thrombosis Outcomes Study investigated whether treatment with canakinumab, a monoclonal antibody that specifically neutralises IL-1b, would benefit people with CRP > 2.0 mg/L and a history of myocardial infarction [6]. As a result, participants who achieved CRP < 2 mg/L showed a 25% reduction in adverse CVD events and a 31% reduction in both all-cause and CVD mortality [6]. Future well-designed and adequately powered treatment trials on CVD outcomes will be essential for any reliable preventive anti-inflammatory therapy, whether dental or pharmacological.

In general, the finding that periodontitis and systemic inflammation considerably interact on various health conditions, including all-cause and cause-specific mortality, has important clinical implications. First, individuals with both periodontitis and systemic inflammation are at significantly higher risk, as evidenced by a relative excess in mortality risk of up to 28% due to additive interaction. Therefore, identifying and treating these individuals may be beneficial in preventing and reducing the serious consequences of inflammation-related disease. Second, the observed additive interaction suggests that interventions aimed at reducing periodontitis and systemic inflammation may also have synergistic effects on health outcomes. Thus, lifestyle modifications, treatments or medications addressing both factors simultaneously may have even greater benefits than targeting each factor separately. Finally, this relationship highlights the importance of interdisciplinary collaboration between medical and dental professionals in the prevention and management of general health.

### Strengths and limitations

To investigate the interaction of periodontitis and systemic inflammation on all-cause and CVD mortality, we analysed 13-year survival data from more than 3000 participants in the population-based prospective SHIP cohort, including extensive follow-up measurements. In addition, compared with previous publications on this topic, the comprehensive DAG-based adjustment and, in particular, the adjustment for time-varying exposures and confounders are major strengths of the present study.

It would have been desirable to include detailed information on the periodontal treatment received in private dental practices between the SHIP measurements. In fact, participants were asked about periodontal treatment in the last 5 years (yes/no) at the follow-up visits (SHIP-START-1/2), but the validity of this information was limited because of participants’ recall and frequent confusion with professional tooth cleaning and also because of the widespread inadequate periodontal treatment in private practices [78]. The same applies to the additional modelling of self-reported non-fatal CVD events. Furthermore, despite the greatest care and review of the underlying model assumptions, there is always a risk of residual confounding and unknown bias. The use of scores for periodontitis and systemic inflammation aggregated different parameters into one value, so that a small increase in several parameters could be represented in the same way as a large increase in a single measure. This could be of great benefit for risk assessment, but without established scores, the comparability of results is clearly limited.

## Conclusions

Periodontitis and systemic inflammation were both significantly associated with all considered types of mortality. On the additive scale, excess risks of 19% and 28% due to interaction were observed for all-cause mortality and CVD mortality, respectively. In contrast, the mediation of effects was marginal and the classical multiplicative interaction analysis was unable to quantify the interplay between periodontitis and inflammation. However, the possibility of risk reduction through anti-inflammatory periodontal treatment seems worthy of further investigation, especially in patients with both periodontitis and high systemic inflammation. Furthermore, the finding of a synergistic effect of periodontitis and systemic inflammation on mortality highlights the need for multidisciplinary approaches to address risk factors and improve health outcomes.

## Supplementary Information

**Additional file 1: Table S1.** Characteristics of the study sample at baseline and follow-up examinations. **Figure S1.** Main directed acyclic graph (DAG) to evaluate the interaction of systemic inflammation and periodontitis on mortality. **Table S2.** Minimal sufficient adjustment sets for estimating possible effects on mortality. **Table S3.** Cut-off values for classification of periodontitis and inflammation in interaction analyses. **Figure S2.** Kaplan-Meier curves for overall survival. **Figure S3.** Survival probability, risk tables and associated events stratified by I-Score, P-Score, CRP and mean CAL. **Figure S4.** Survival probabilities, risk tables and associated events stratified by further inflammation markers and periodontitis measures. **Figure S5.** Observed crude incidence rate ratios representing the interaction of further inflammation markers and periodontitis measures regarding all-cause mortality (A-H). **Figure S6.** Observed crude incidence rate ratios representing the interaction of inflammation and periodontitis regarding all-cause mortality in never smoker only, *n* = 1081. **Table S4.** Effects of periodontitis and systemic inflammation measures on all-cause, CVD and non-CVD mortality. **Table S5.** Interaction of periodontitis and systemic inflammation regarding mortality: overview and ranking. **Table S6.** Sample analytic code.

## Data Availability

All SHIP data are the legal property of the University Medicine Greifswald, represented by the steering committee of the Community Medicine Research Network. Because of the extremely comprehensive information collected from a considerable proportion of the regional population, the SHIP data are not freely accessible. For scientific purposes, there is a standardised procedure for the use and access to all data and biomaterials. Please visit https://transfer.ship-med.uni-greifswald.de/FAIRequest for further information and requests for use of SHIP data.

## Abbreviations

^18^F-FDG-PET/CT: ^18^F-fluorodeoxyglucose positron emission tomography/computed tomography
AAP: American Academy of Periodontitis
ATC: Anatomical Therapeutic Chemical Classification System
BMI: Body mass index
CAL: Clinical attachment level
CDC: Centres for Disease Control and Prevention
CI: Confidence interval
CRP: C-reactive protein
CVD: Cardiovascular disease
DAG: Directed acyclic graph
HDL-C: High-density lipoprotein cholesterol
HR: Hazard ratio
I-Score: Inflammation score
ICD10: International Classification of Diseases, 10th revision
IRB: Integrated Research Biobank
IRR: Incidence rate ratio
LDL-C: Low-density lipoprotein cholesterol
P-Score: Periodontitis score
PPD: Pocket probing depth
PRIME: Prospective Epidemiological Study of Myocardial Infarction
RERI: Relative excess risk due to interaction
RR: Relative risk
SD: Standard deviation
SHIP: Study of Health in Pomerania
WHO: World Health Organization
