# Correction: Periodontitis and systemic inflammation as independent and interacting risk factors for mortality: evidence from a prospective cohort study

**DOI:** 10.1186/s12916-025-03862-0

**Published:** 2025-01-15

**Authors:** Christiane Pink, Birte Holtfreter, Henry Völzke, Matthias Nauck, Marcus Dörr, Thomas Kocher

**Affiliations:** 1 Department of Restorative Dentistry, Periodontology, Endodontology and Preventive and Pediatric Dentistry, University Medicine Greifswald, Fleischmannstr. 42, 17475 Greifswald, Germany; 2 Department of Orthodontics, University Medicine Greifswald, Greifswald, Germany; 3 German Centre for Cardiovascular Research (DZHK), Partner Site Greifswald, Greifswald, Germany; 4 Institute for Community Medicine, SHIP/Clinical-Epidemiological Research, University Medicine Greifswald, Greifswald, Germany; 5 Institute of Clinical Chemistry and Laboratory Medicine, University Medicine Greifswald, Greifswald, Germany; 6 Department of Internal Medicine B, University Medicine Greifswald, Greifswald, Germany

**Correction** **:** **BMC Med 21, 430 (2023)**

**https://doi.org/10.1186/s12916-023-03139-4**

The authors wish to note the following Funding source which was mistakenly omitted from the original article [1]:

‘CP was funded by the Deutsche Forschungsgemeinschaft (DFG, German Research Foundation) - 451892213.’
