# Supplementary material for: Periodontitis and systemic inflammation as independent and interacting risk factors for mortality: evidence from a prospective cohort study
Source: BMC Med. 2023 Nov 13;21:430. doi: 10.1186/s12916-023-03139-4 (PMC10642059; doi:10.1186/s12916-023-03139-4)
Supplement: Supplementary file 1 — Additional file 1: Table S1. Characteristics of the study sample at baseline and follow-up examinations. Figure S1. Main directed acyclic graph (DAG) to evaluate the interaction of systemic inflammation and periodontitis on mortality. Table S2. Minimal sufficient adjustment sets for estimating possible effects on mortality. Table S3. Cut-off values for classification of periodontitis and inflammation in interaction analyses. Figure S2. Kaplan-Meier curves for overall survival. Figure S3. Survival probability, risk tables and associated events stratified by I-Score, P-Score, CRP and mean CAL. Figure S4. Survival probabilities, risk tables and associated events stratified by further inflammation markers and periodontitis measures. Figure S5. Observed crude incidence rate ratios representing the interaction of further inflammation markers and periodontitis measures regarding all-cause mortality (A-H). Figure S6. Observed crude incidence rate ratios representing the interaction of inflammation and periodontitis regarding all-cause mortality in never smoker only, n = 1081. Table S4. Effects of periodontitis and systemic inflammation measures on all-cause, CVD and non-CVD mortality. Table S5. Interaction of periodontitis and systemic inflammation regarding mortality: overview and ranking. Table S6. Sample analytic code. [file 12916_2023_3139_MOESM1_ESM.docx]

**Periodontitis and systemic inflammation as independent and interacting risk factors for mortality**

**- Supplementary information -**

**Table S1.** Characteristics of the study sample at baseline and follow-up examinations.

|  | **Complete sample** | **SHIP-START-1 subsample*** | | | **SHIP-START-2 subsample*** | | |
| --- | --- | --- | --- | --- | --- | --- | --- |
|  | **SHIP-**  **START-0** | **SHIP- START-0** | **SHIP- START-1** | **P-Value** | **SHIP- START-0** | **SHIP- START-2** | **P-Value** |
| N | 3047 | 2421 | 2421 | - | 1852 | 1852 | - |
| Observation period, years | 13.0 ± 2.4 | 13.4 ± 1.5 | 13.4 ± 1.5 | - | 13.6 ± 1.1 | 13.6 ± 1.1 | - |
| Sex (male) | 1486 (48.8) | 1159 (47.9) | 1159 (47.9) | - | 862 (46.5) | 862 (46.5) | - |
| Age, years | 46.6 ± 15.2 | 46.8 ± 14.3 | 52.0 ± 14.3 | <0.001^a^ | 45.1 ± 13.3 | 56.2 ± 13.1 | <0.001^a^ |
| Living in a partnership, yes | 2348 (77.1) | 1913 (79.0) | 1882 (77.7) | 0.07^b^ | 1486 (80.2) | 1468 (79.3) | 0.33^b^ |
| Equivalised income |  |  |  | <0.001^c^ |  |  | <0.001^c^ |
| 1^st^ tertile, <750 € | 1019 (33.4) | 743 (30.7) | 1083 (44.7) |  | 544 (29.4) | 732 (39.5) |  |
| 2^nd^ tertile, 750-1200 € | 1170 (38.4) | 937 (38.7) | 602 (24.9) |  | 696 (37.6) | 531 (28.7) |  |
| 3^rd^ tertile, >1200 € | 858 (28.2) | 741 (30.6) | 736 (30.4) |  | 612 (33.0) | 589 (31.8) |  |
| Smoking status |  |  |  | 0.22^c^ |  |  | <0.001^c^ |
| Never smoker | 1081 (35.5) | 901 (37.2) | 861 (35.6) |  | 715 (38.6) | 637 (34.4) |  |
| Former smoker | 990 (32.5) | 815 (33.7) | 900 (37.2) |  | 615 (33.2) | 832 (44.9) |  |
| Current smoker | 976 (32.0) | 705 (29.1) | 660 (27.3) |  | 522 (28.2) | 383 (20.7) |  |
| Body Mass Index |  |  |  | <0.001^c^ |  |  | <0.001^c^ |
| <25 kg/m^2^ | 1115 (36.6) | 881 (36.4) | 754 (31.1) |  | 712 (38.4) | 510 (27.5) |  |
| 25-<30 kg/m^2^ | 1208 (39.7) | 970 (40.1) | 957 (39.5) |  | 737 (39.8) | 741 (40.0) |  |
| ≥30 kg/m^2^ | 742 (23.8) | 570 (23.5) | 710 (29.3) |  | 403 (21.8) | 601 (32.5) |  |
| Diabetes mellitus, yes | 250 (8.2) | 180 (7.4) | 248 (10.2) | <0.001^b^ | 100 (5.4) | 235 (12.7) | <0.001^b^ |
| Physical activity, yes | 1406 (46.1) | 1129 (46.6) | 904 (37.3) | <0.001^b^ | 909 (49.1) | 1261 (68.1) | <0.001^b^ |
| Dyslipidaemia, yes | 1415 (46.4) | 1132 (46.8) | 1524 (63.0) | <0.001^b^ | 813 (43.9) | 939 (50.7) | <0.001^b^ |
| High alcohol consumption, yes | 446 (14.6) | 364 (15.0) | 238 (9.8) | <0.001^b^ | 285 (15.4) | 191 (10.3) | <0.001^b^ |
| Dental check-up during the last year, yes | 2729 (89.6) | 2194 (90.6) | 2244 (92.7) | 0.002^b^ | 1697 (91.6) | 1708 (92.2) | 0.49^b^ |
| Daily tooth brushing frequency ≥2 | 2531 (83.1) | 2032 (83.9) | -** | - | 1575 (85.0) | 1599 (86.3) | 0.14^b^ |
| Mean PPD, mm | 2.5 ± 0.7 | 2.5 ± 0.7 | 2.4 ± 0.7 | <0.001^a^ | 2.4 ± 0.6 | 2.6 ± 0.6 | <0.00 ^a^ |
| Percentage of sites with PPD ≥3 mm, % | 44.9 ± 24.2 | 44.5 ± 23.7 | -** | - | 41.3 ± 22.2 | 51.9 ± 22.1 | <0.001^a^ |
| Percentage of sites with PPD ≥4 mm, % | 12.3 ± 16.9 | 11.9 ± 16.4 | -** | - | 9.5 ± 13.4 | 13.3 ± 16.4 | <0.001^a^ |
| Mean CAL, mm | 2.6 ± 1.9 | 2.5 ± 1.7 | 2.6 ± 1.9 | 0.003^a^ | 2.4 ± 1.5 | 2.9 ± 1.7 | <0.001^a^ |
| Percentage of sites with CAL ≥3 mm, % | 46.0 ± 34.8 | 45.5 ± 34.0 | -** | - | 40.4 ± 32.4 | 51.7 ± 34.4 | <0.001^a^ |
| Percentage of sites with CAL ≥4 mm, % | 27.4 ± 31.7 | 26.5 ± 30.7 | -** | - | 21.2 ± 26.7 | 30.5 ± 32.0 | <0.001^a^ |
| Number of missing teeth | 7.1 ± 6.8 | 6.8 ± 6.6 | 7.7 ± 7.4 | <0.001^a^ | 5.9 ± 5.9 | 7.6 ± 7.4 | <0.001^a^ |
| Periodontitis score | 0.0 ± 0.9 | -0.1 ± 0.9 | 0.0 ± 0.9 | <0.001^a^ | -0.2 ± 0.8 | 0.0 ± 0.9 | <0.001^a^ |
| Leukocytes, Gpt/l | 6.7 ± 2.0 | 6.6 ± 1.9 | 6.9 ± 1.9 | <0.001^a^ | 6.6 ± 1.9 | 6.2 ± 1.8 | <0.001^a^ |
| Fibrinogen, g/l | 2.9 ± 0.7 | 2.9 ± 0.7 | 3.2 ± 0.8 | <0.001^a^ | 2.9 ± 0.6 | 3.1 ± 0.8 | <0.001^a^ |
| C-Reactive Protein, mg/l | 2.7 ± 5.4 | 2.5 ± 4.3 | 2.8 ± 5.6 | 0.04^a^ | 2.5 ± 4.4 | 2.0 ± 2.4 | <0.001^a^ |
| Inflammation score | 0.0 ± 0.8 | 0.0 ± 0.7 | 0.0 ± 0.7 | 0.01^a^ | -0.1 ± 0.7 | 0.0 ± 0.8 | <0.001^a^ |

Data are presented as mean ± standard deviation or number (percentage). Abbreviations: CVD, cardiovascular disease; PPD, pocket probing depth; CAL, clinical attachment level.

Equivalised income: net household income divided by the square root of household size. Diabetes mellitus: self-reported physician’s diagnosis or antidiabetic treatment or non-fasting glucose levels ≥11.1 mmol/l or glycated haemoglobin concentrations ≥48 mmol/mol. Physical activity: at least 1 h of physical exercise per week during summer or winter. Dyslipidaemia: total cholesterol ≥6.2 mmol/l, low density lipoprotein cholesterol ≥4.1 mmol/l, high density lipoprotein cholesterol <1.04 mmol/l or use of lipid-modifying agents. High alcohol consumption: average pure ethanol intake of ≥30 g per day for men and ≥20 g per day for women. Periodontitis score: mean of z-transformed values for missing teeth, mean CAL and percentage of sites with CAL ≥ 3mm. Inflammation score: mean of z-transformed values for leukocytes, fibrinogen and C-reactive protein.

P-values were obtained using paired t-tests (continuous variables: a), McNemar's tests (dichotomous variables: b) and Wilcoxon signed rank tests (ordinal variables: c).

* All SHIP-START-0 participants were re-invited for SHIP-START-2 irrespective of interim SHIP-START-1 participation. Thus, SHIP-START-2 is not a proper subset of SHIP-START-1 and therefore not compared to SHIP-START-1 but to SHIP-START-0.

** In SHIP-START-1, the dental examination protocol, including the periodontal probe used to measure PPD and CAL, was changed (SHIP-START-0 /2: PCP11, SHIP-START-1: PCP2, Hu-Friedy, Chicago, IL, USA). As the probe change resulted in biased estimates of changes in PPD and CAL due to digit preference effects, SHIP-START-1 data were transformed according to correction values obtained from a cross-over study [29]. This resulted in: i) probe-corrected mean values for PPD and CAL but ii) no percentages of sites with PPD/CAL ≥3 mm or 4 mm in SHIP-START-1. In addition, the SHIP-START-1 dental examination protocol did not include the collection of information on brushing frequency. For SHIP-START-2, the original SHIP-START-0 examination protocol was reinstated.

**Figure S1.** Main directed acyclic graph (DAG) to evaluate the interaction of systemic inflam-mation and periodontitis on mortality.

In case of no significant struc-tural changes to the graph, multiple variables were conden-sed to combined nodes (e.g. age and sex) for enhanced clarity in the graphical representation.

Minimal sufficient adjustment sets resulting from DAG analyses are presented in Table S2.


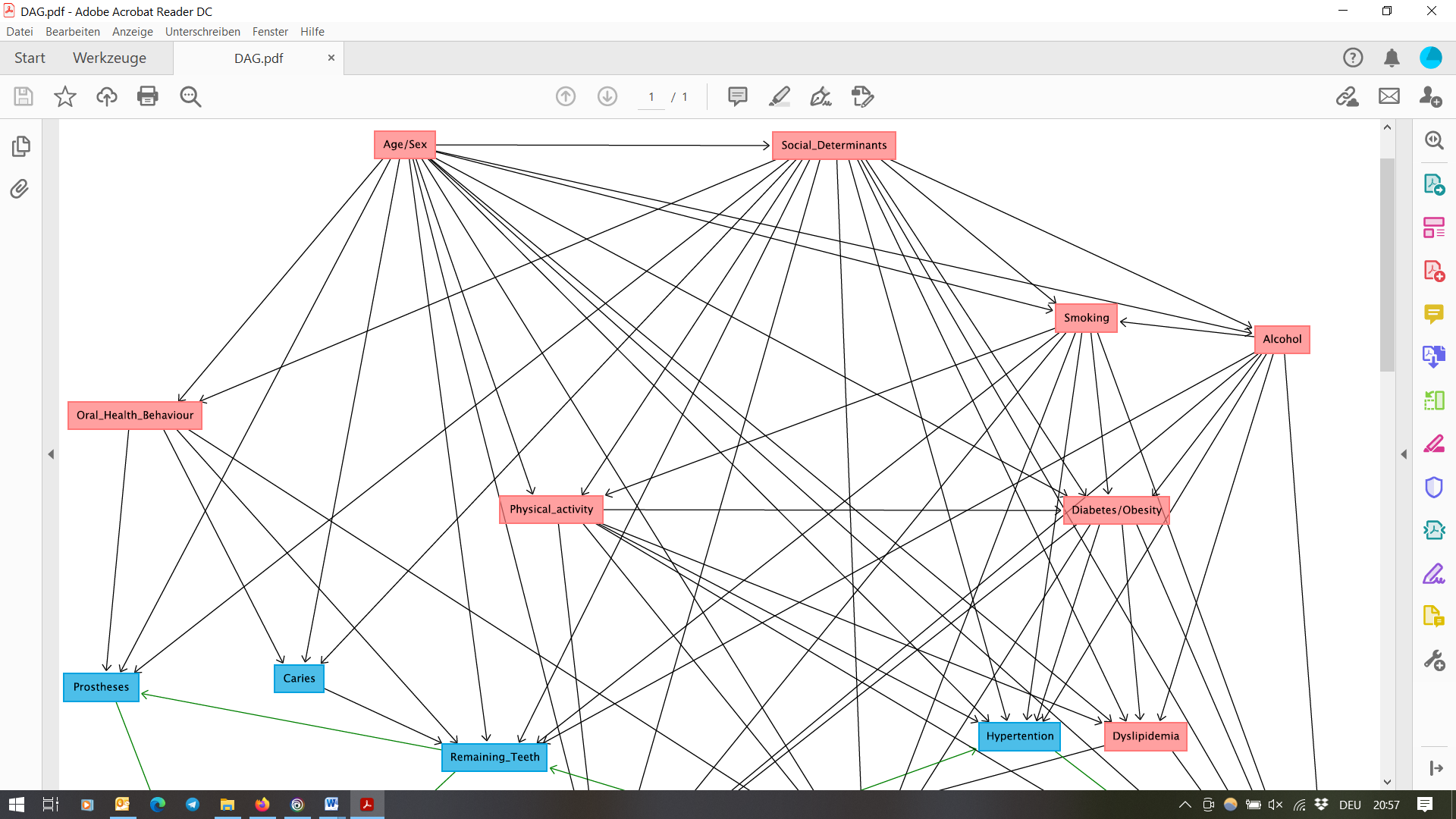

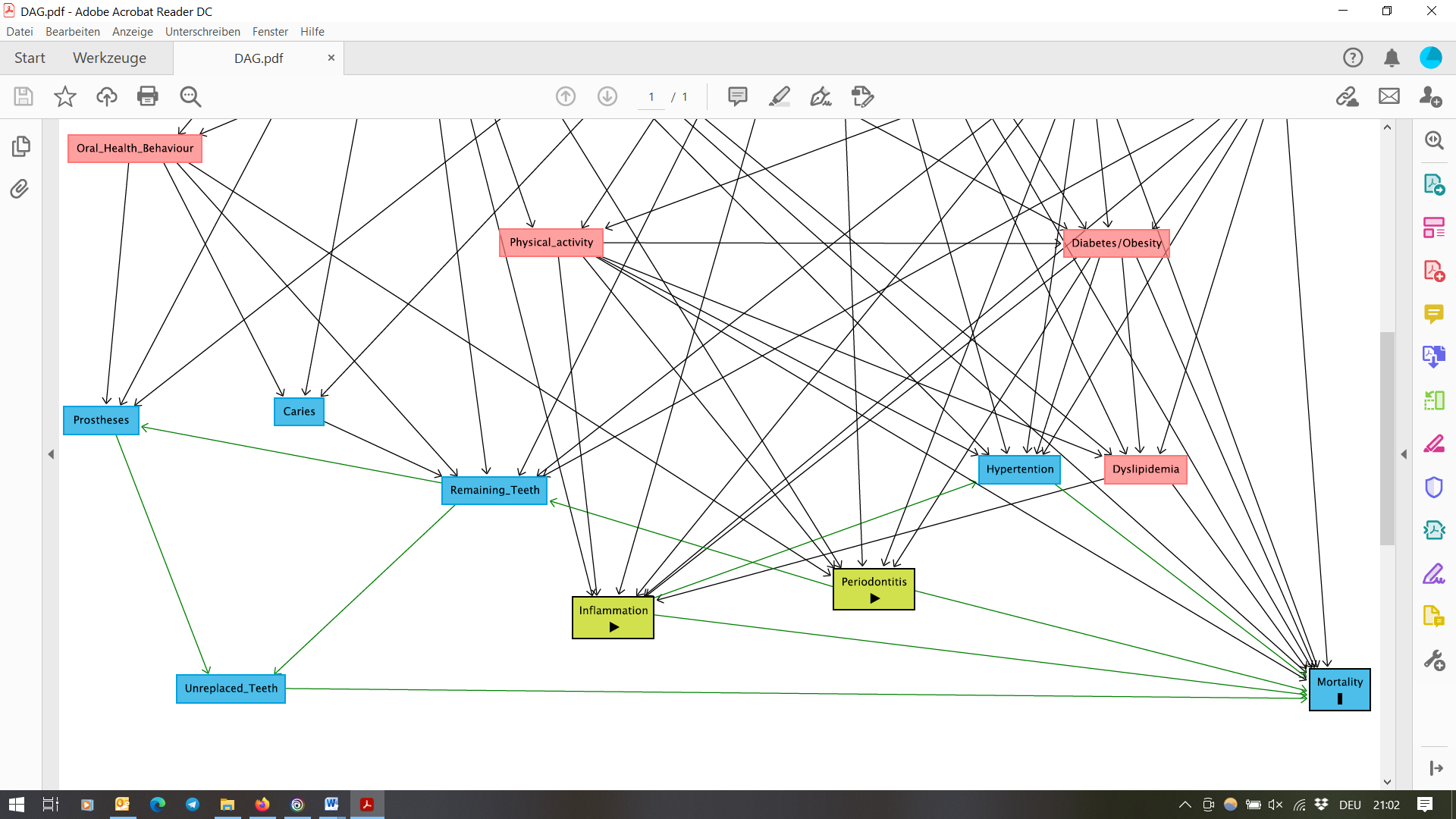


Symbols and colours:

- Exposure
- Outcome
- Ancestor of exposure
- Ancestor of outcome
- Ancestor of exposure

and outcome

**Table S2.** Minimal sufficient adjustment sets for estimating possible effects on mortality.

|  | **Exposure** | | |
| --- | --- | --- | --- |
| **Covariate** | **Inflammation** | **Periodontitis** | **Both*** |
| Age | X | X | X |
| Sex | X | X | X |
| Living in a partnership | X | X | X |
| Equivalised Income | X | X | X |
| Smoking status | X | X | X |
| Body Mass Index | X | X | X |
| Diabetes mellitus | X | X | X |
| Physical Activity | X | X | X |
| Alcohol consumption | X |  | X |
| Dyslipidaemia | X |  | X |
| Dental check-ups |  | X | X |
| Tooth brushing frequency |  | X | X |

DAGs often provide more than one minimal sufficient adjustment set. However, the adjustment sets listed above represent the most robust options in terms of inclusion/exclusion of arrows with limited evidence or uncertain direction.

*Both exposures are included in the same model in case of interaction and mediation analyses. This constellation and minimal sufficient adjustment set correspond to the directed acyclic graph as shown in Figure S1.

**Table S3.** Cut-off values for classification of periodontitis and inflammation in interaction analyses.

| **Periodontitis measure** | **Cut-off** | **N of low / high category** | **Mean of low / high category** |
| --- | --- | --- | --- |
| Mean PPD, mm | ≥ 2.6 | 2008 / 1039 | 2.10 / 3.25 |
| Percentage of sites with PPD≥3mm, % | ≥ 55 | 1998 / 1049 | 30.51 / 72.30 |
| Percentage of sites with PPD≥4mm, % | ≥ 10 | 1920 / 1127 | 2.54 / 28.82 |
| Mean CAL, mm | ≥ 3.0 | 2005 / 1042 | 1.52 / 4.68 |
| Percentage of sites with CAL≥3mm, % | ≥ 68 | 2045 / 1002 | 25.13 / 88.45 |
| Percentage of sites with CAL≥4mm, % | ≥ 34 | 2055 / 992 | 7.80 / 68.06 |
| Number of missing teeth | ≥ 8 | 2077 / 970 | 3.14 / 15.59 |
| Periodontitis Score | ≥ 0.3 | 2030 / 1017 | -0.55 / 1.10 |
|  |  | | |
| **Systemic Inflammation measure** | **Cut-off** | **N of low / high category** | **Mean of low / high category** |
| Leukocytes, Gpt/l | ≥ 7.3 | 2040 / 1007 | 5.66 / 8.87 |
| Fibrinogen, g/l | ≥ 3.0 | 1805 / 1242 | 2.50 / 3.57 |
| C-Reactive Protein, mg/l | ≥ 2.7 | 2243 / 804 | 1.02 / 7.42 |
| Inflammation score | ≥ 0.3 | 2111 / 936 | -0.39 / 0.89 |

Cut-off values were chosen to divide the study population into approximately one upper third (high category) and two lower thirds (low category) for each measure of periodontitis and systemic inflammation, while ensuring a sufficient distribution of subjects across strata when considering all possible pairs of measures in interaction analyses.

Abbreviations: PPD, pocket probing depth; CAL, clinical attachment level.


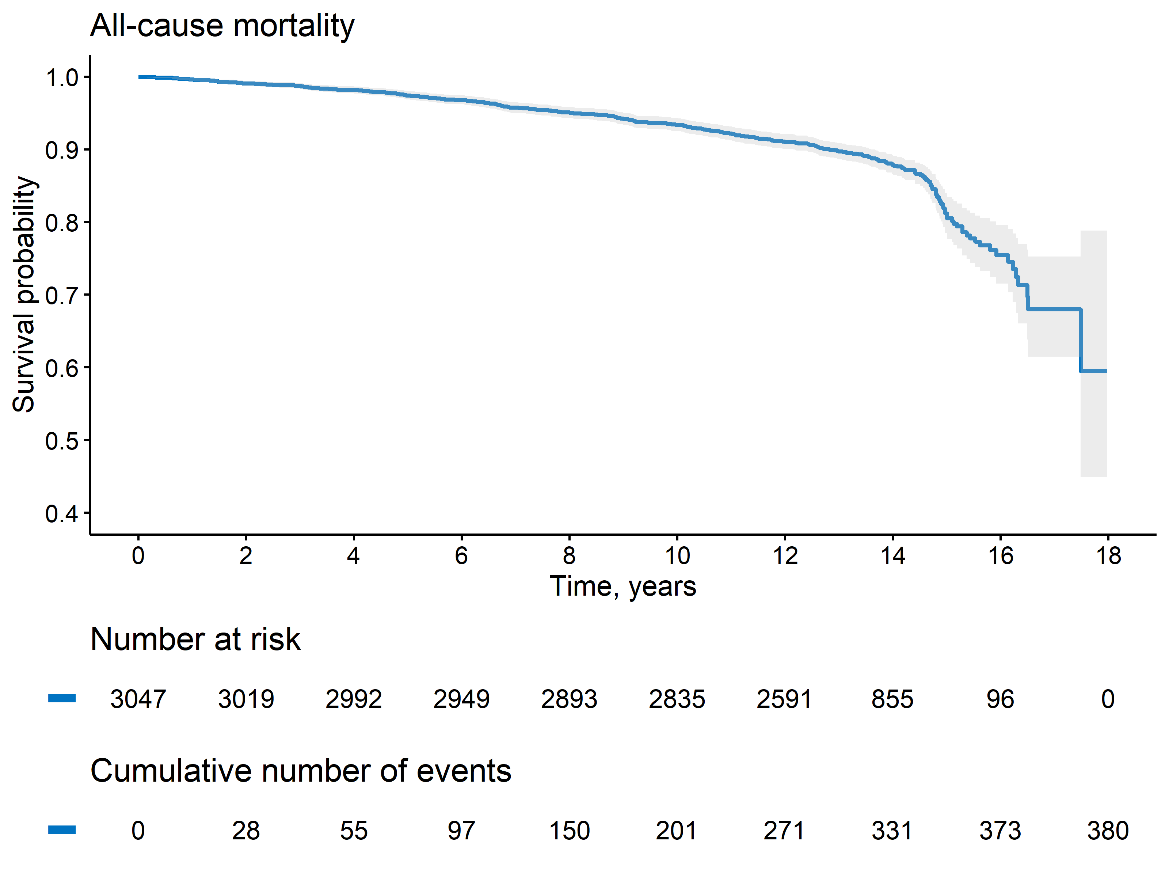


A


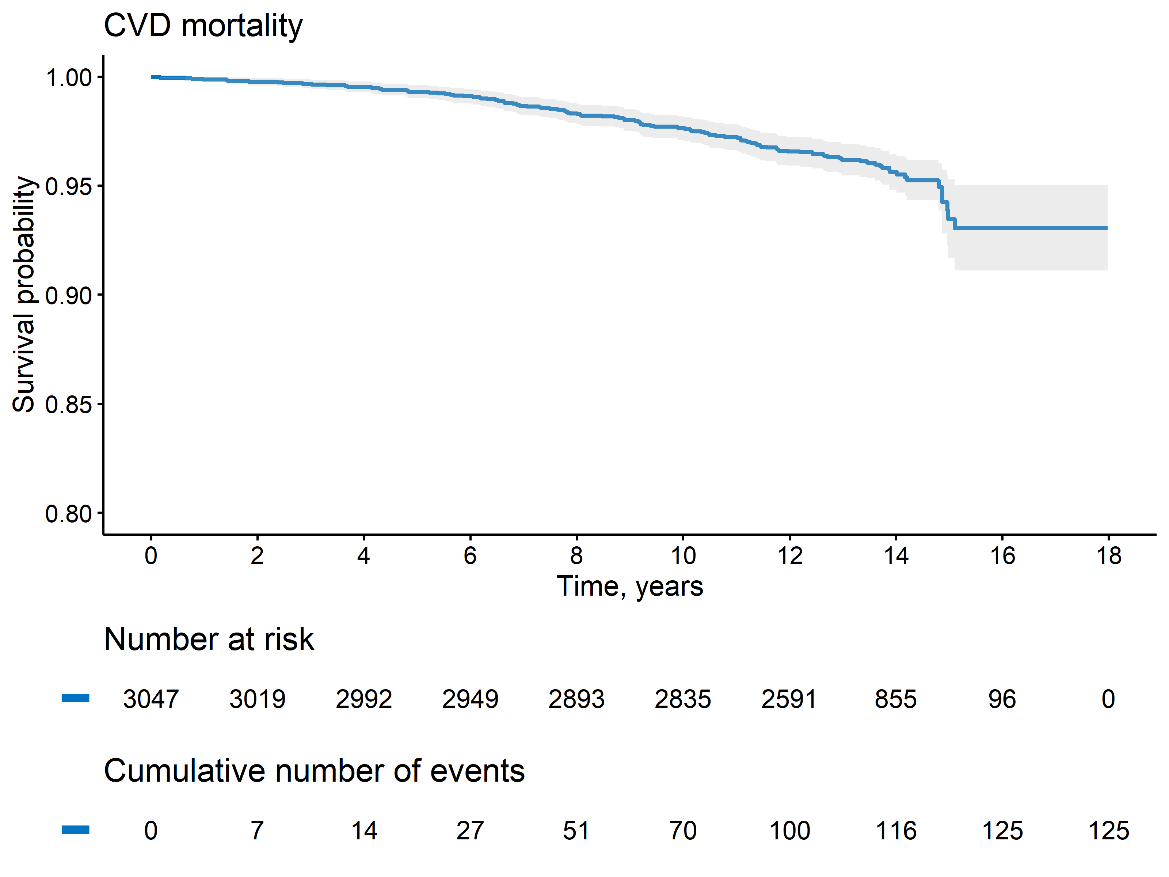


B


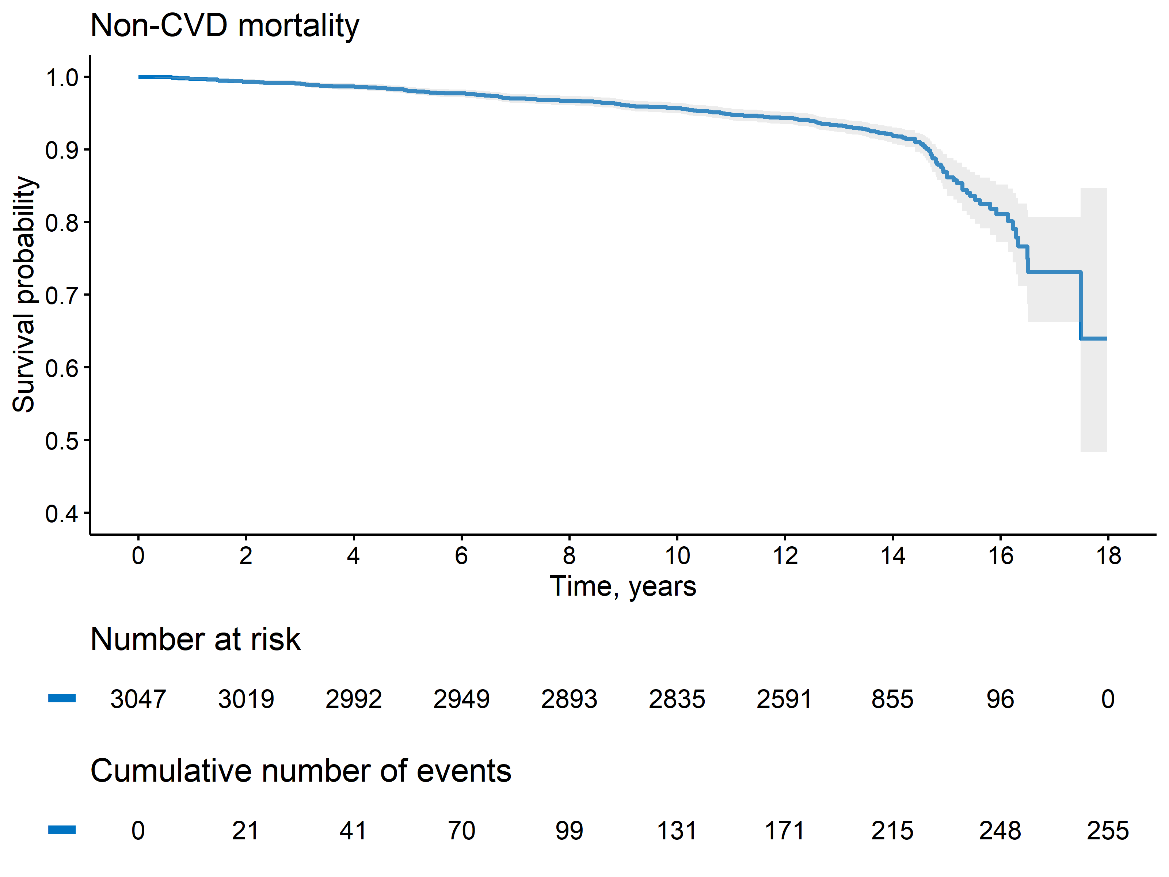


C

**Figure S2.** Kaplan-Meier curves for overall survival.

Overall survival probabilities of study participants regarding all-cause mortality (A), CVD mortality (B) and non-CVD mortality (C).

Abbreviations: CVD, cardiovascular disease.


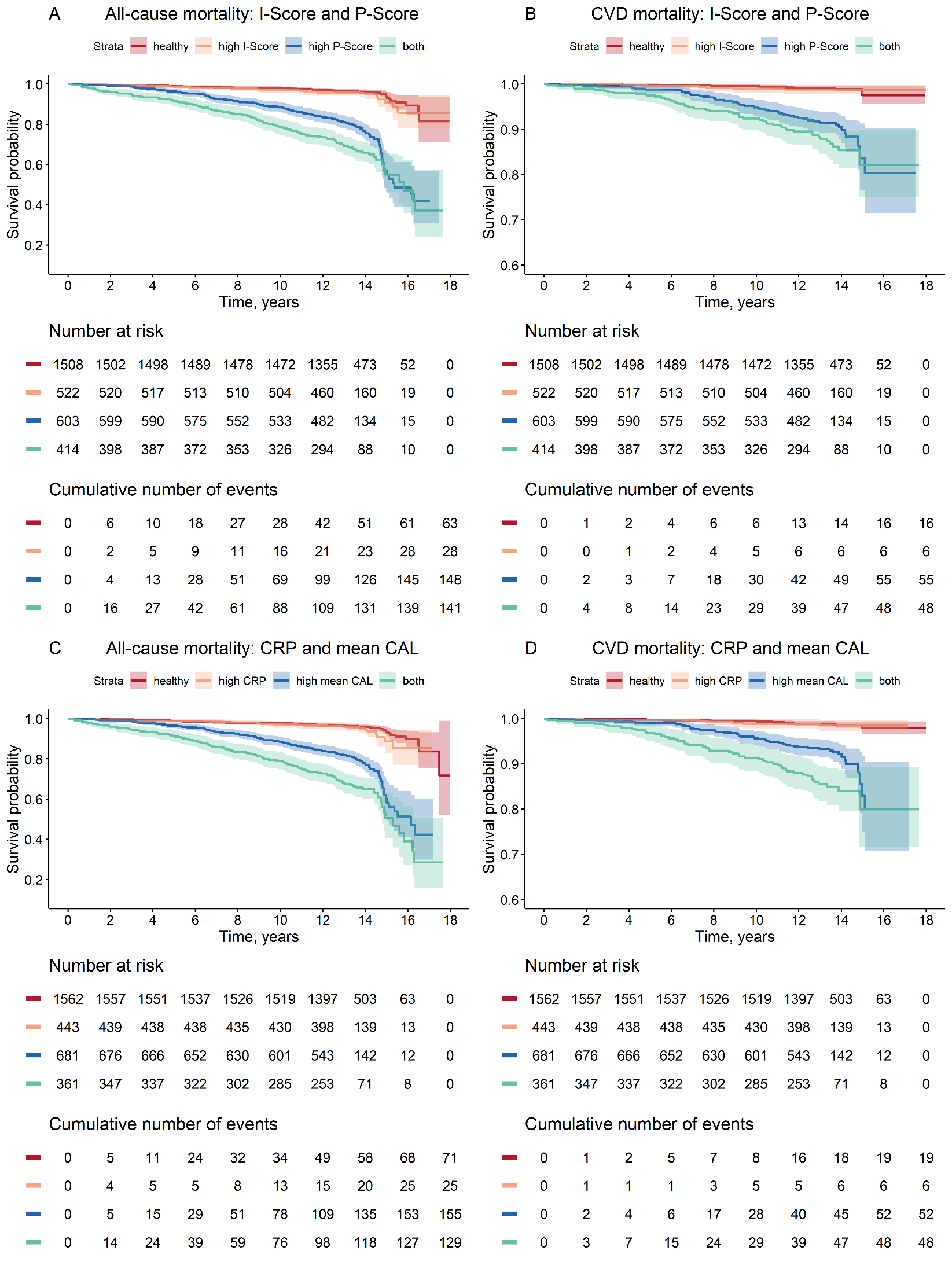


**Figure S3.** Survival probability, risk tables and associated events stratified by I-Score, P-Score, CRP and mean CAL.

Survival probabilities of study participants regarding all-cause mortality according to inflammation score and periodontitis score (A), CVD mortality according to inflammation score and periodontitis score (B), all-cause mortality according to CRP and mean CAL (C) and CVD mortality according to CRP and mean CAL (D). Underneath the graphical display of Kaplan-Meier curves, the numbers of subjects at risk as well as the cumulative numbers of events are given for all strata. The exact cut-off values for defining the strata are given in Table S3.

Abbreviations: I-Score, inflammation score; P-Score, periodontitis score; CVD, cardiovascular disease; CRP, C-reactive protein; CAL, clinical attachment level.


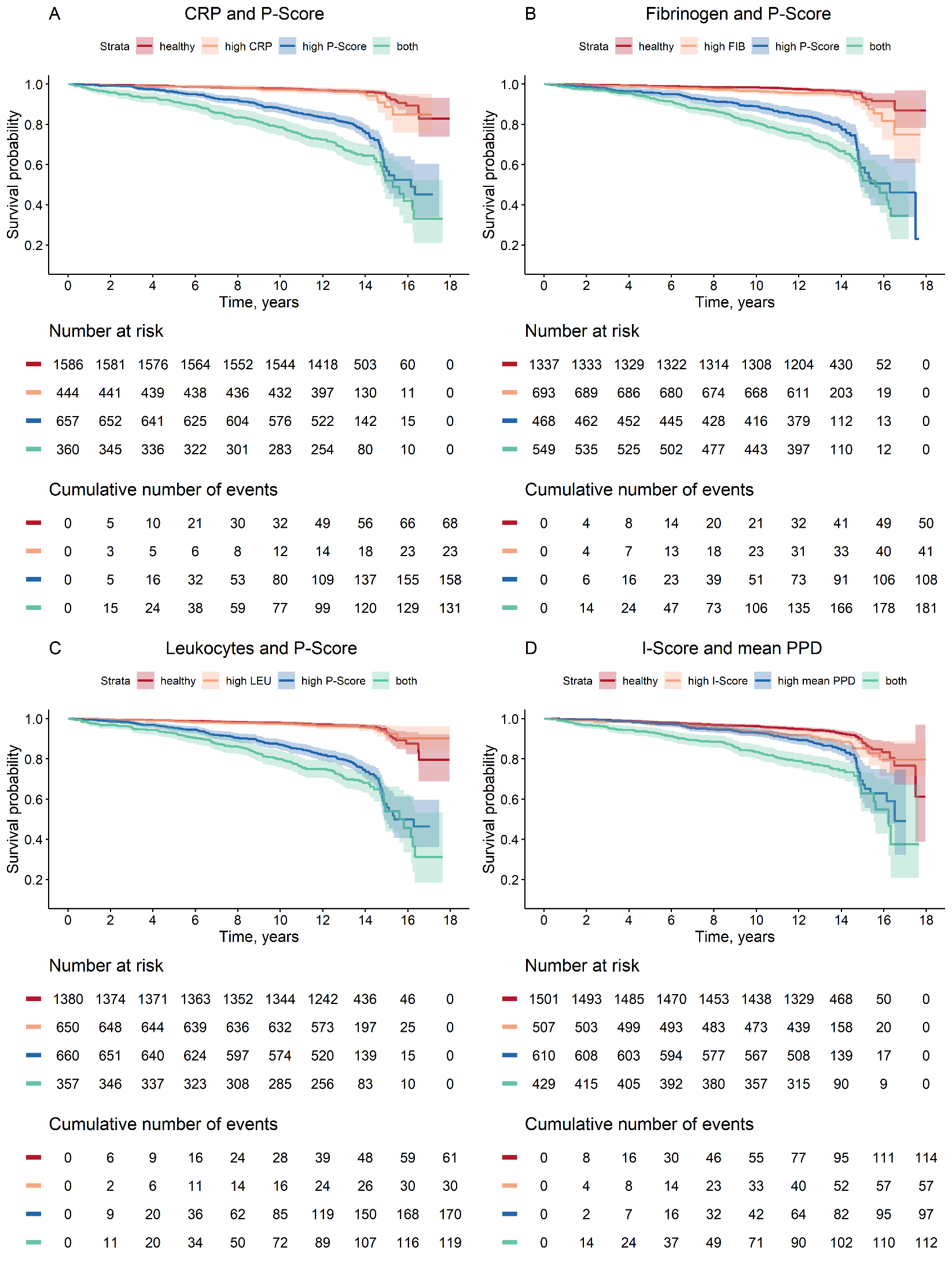


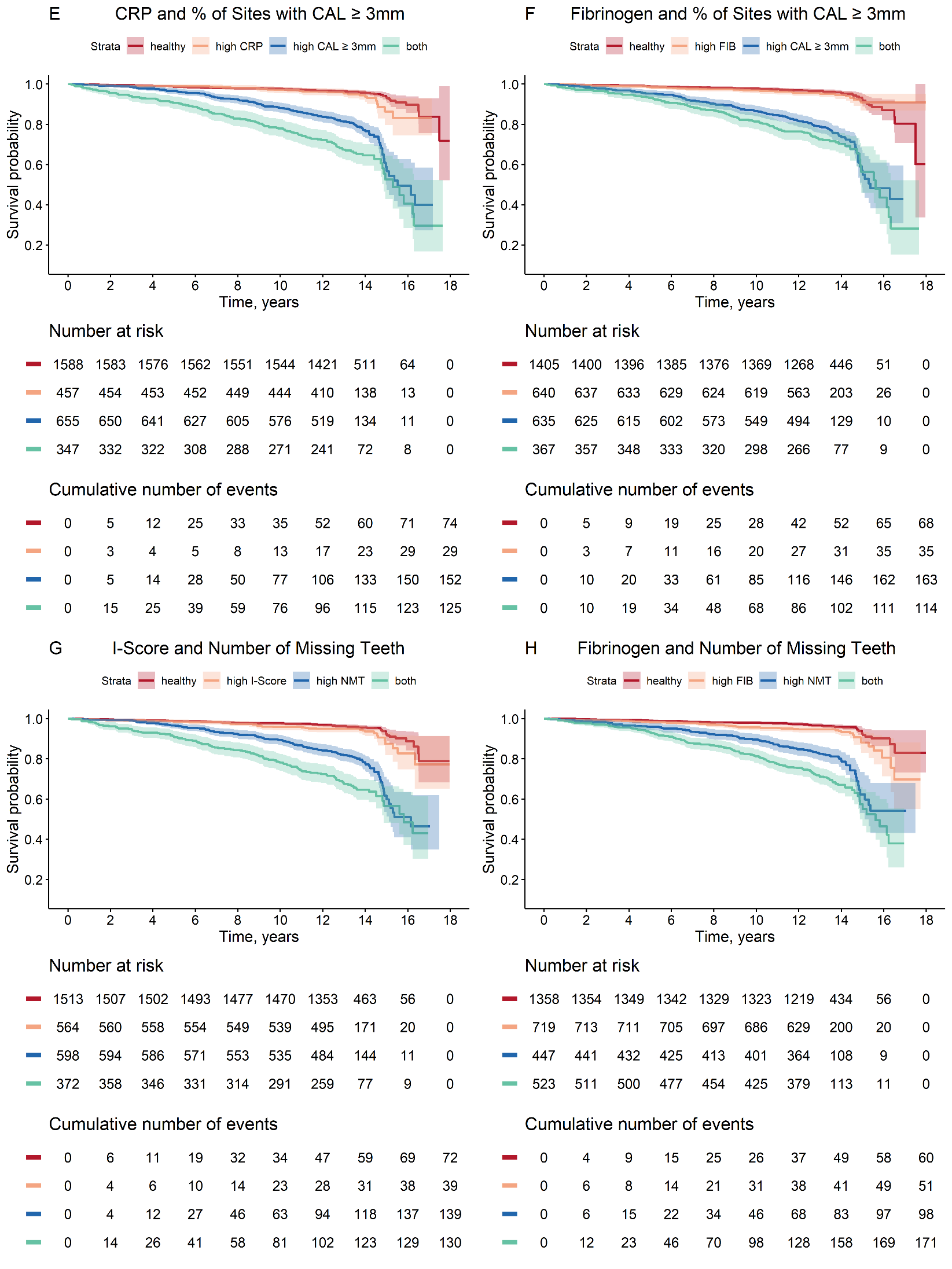


**Figure S4.** Survival probabilities, risk tables and associated events stratified by further inflammation markers and periodontitis measures.

Survival probabilities of study participants regarding all-cause mortality according to C-reactive protein and periodontitis score (A), Fibrinogen and periodontitis score (B), leukocytes and periodontitis score (C), inflammation score and mean PPD (D), C-reactive protein and percentage of sites with CAL ≥ 3mm (E), fibrinogen and percentage of sites with CAL ≥ 3mm (F), inflammation score and number of missing teeth (G) and fibrinogen and number of missing teeth (H). Underneath the graphical display of Kaplan-Meier curves, the numbers of subjects at risk as well as the cumulative numbers of events are given for all strata. The exact cut-off values for defining the strata are given in Table S3.

Abbreviations: I-Score, inflammation score; P-Score, periodontitis score; CRP, C-reactive protein; FIB, fibrinogen; LEU, leukocytes; CAL, clinical attachment level; PPD, pocket probing depth; NMT, number of missing teeth.


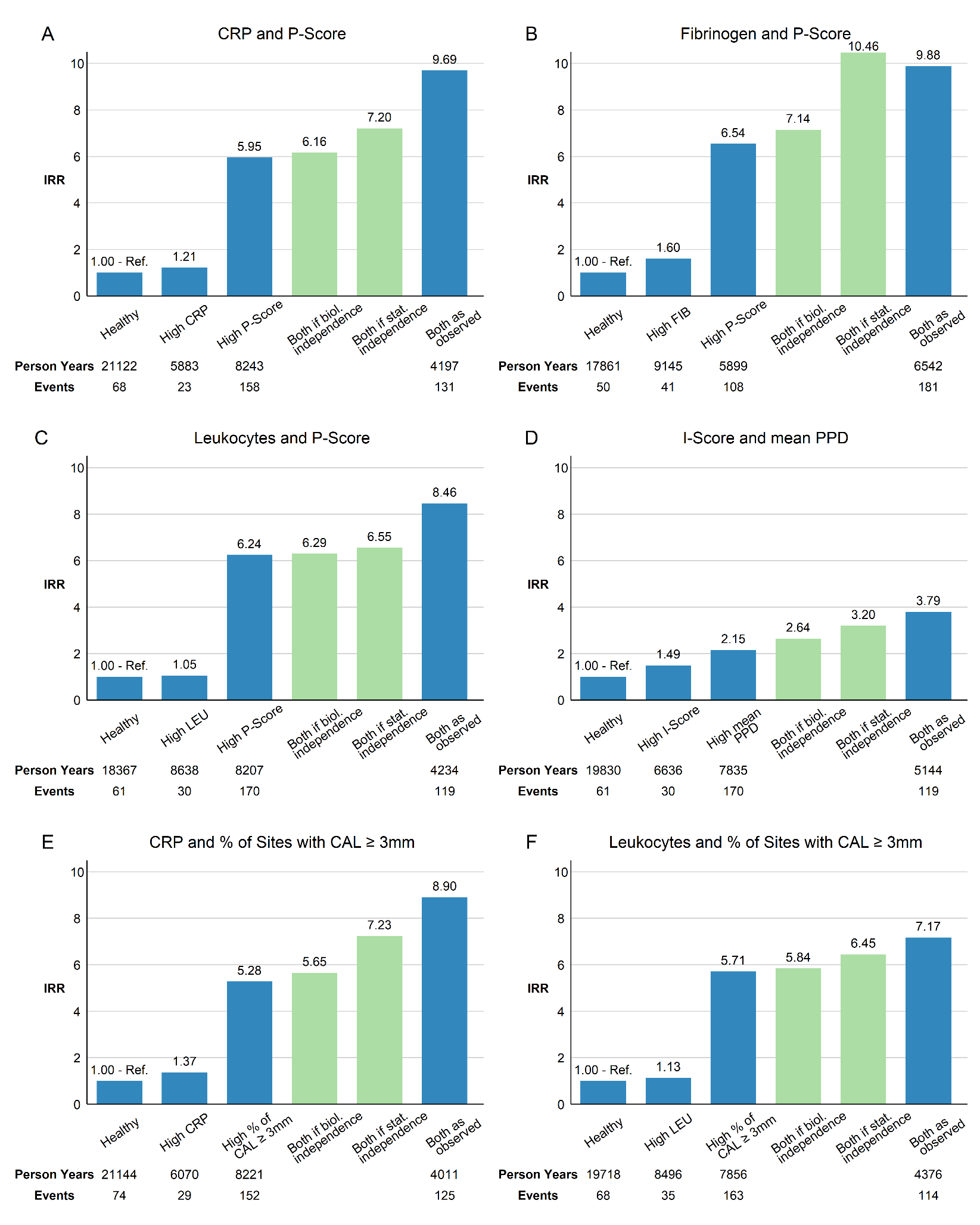


Continued on next page.


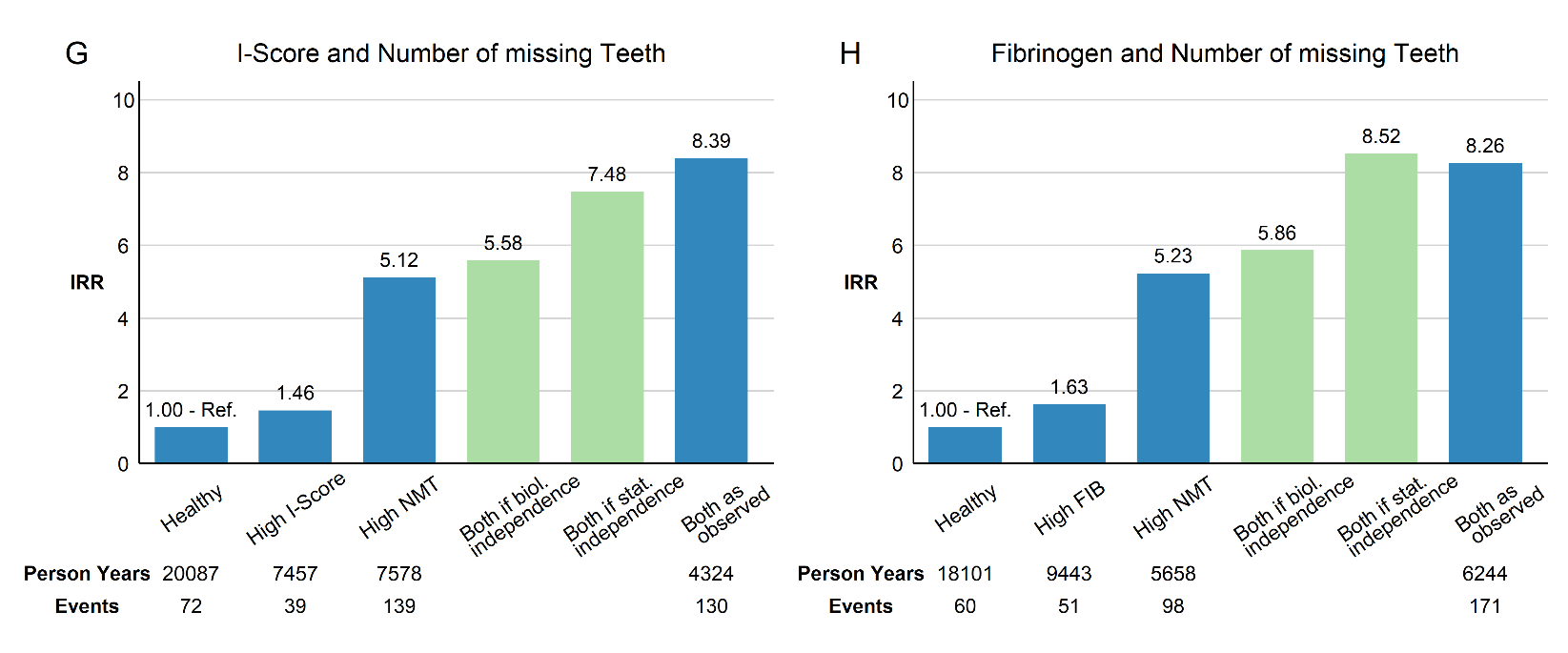
**Figure S5.** Observed crude incidence rate ratios representing the interaction of further inflammation markers and periodontitis measures regarding all-cause mortality (A-H).

Blue bars represent observed IRRs whereas green bars represent the expected IRRs for the strata having high measures for both, inflammation and periodontitis, in case of biological (additive scale) or statistical (multiplicative scale) independence of effects. The exact cut-off values for defining the strata are given in Table S3.

Abbreviations: IRR, incidence rate ratio; I-Score, inflammation score; P-Score, periodontitis score; CRP, C-reactive protein; FIB, fibrinogen; LEU, leukocytes; CAL, clinical attachment level; PPD, pocket probing depth; NMT, number of missing teeth.


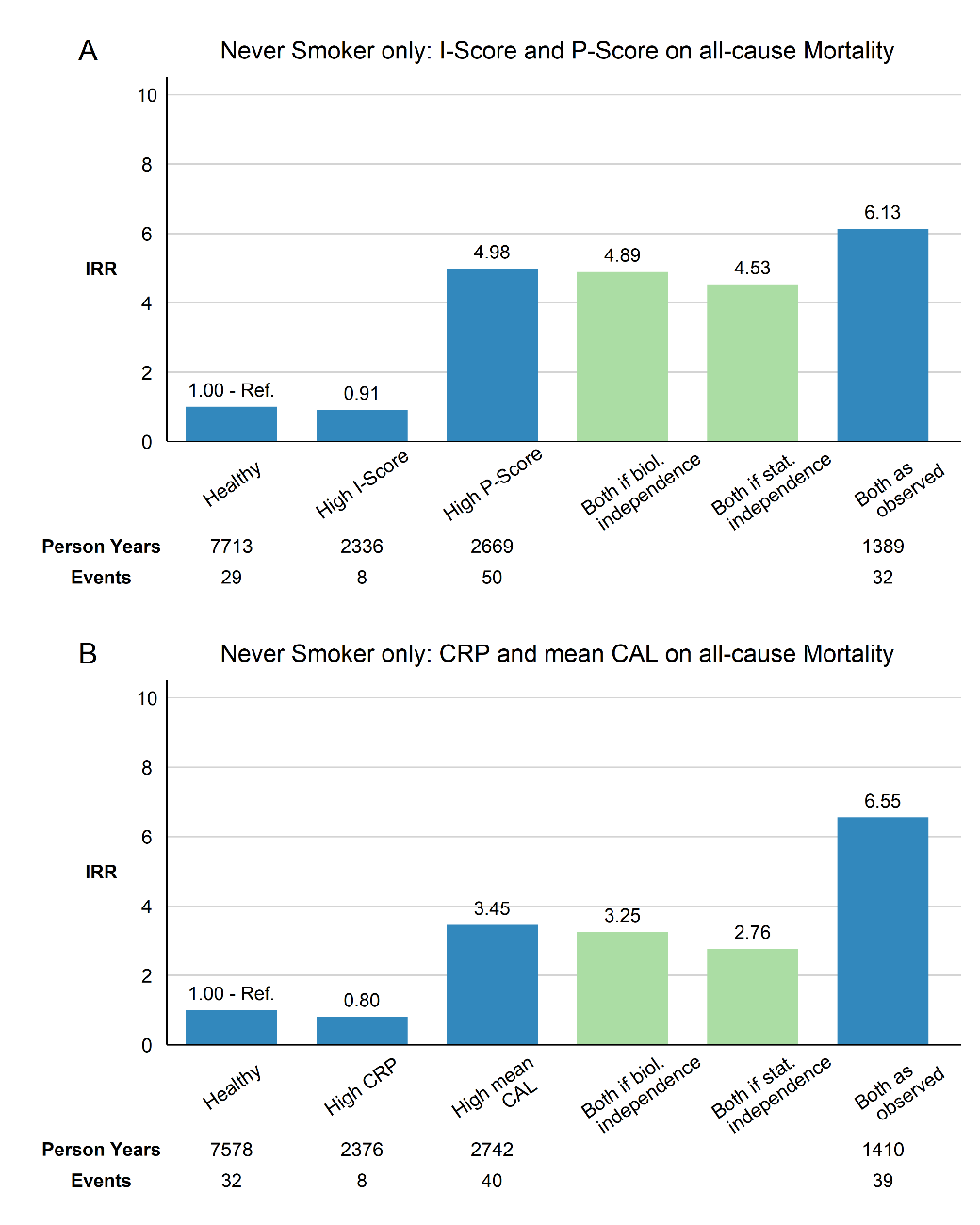


**Figure S6.** Observed crude incidence rate ratios representing the interaction of inflammation and periodontitis regarding all-cause mortality in never smoker only, n=1081.

Blue bars represent observed IRRs whereas green bars represent the expected IRRs for the strata having high measures for both, inflammation and periodontitis, in case of biological (additive scale) or statistical (multiplicative scale) independence of effects. The exact cut-off values for defining the strata are given in Table S3.

Abbreviations: IRR, incidence rate ratio; I-Score, inflammation score; P-Score, periodontitis score; CRP, C-reactive protein; CAL, clinical attachment level.

**Table S4.** Effects of periodontitis and systemic inflammation measures on all-cause, CVD and non-CVD mortality.

| **Periodontitis ^*^** |  | | | | | | | | |
| --- | --- | --- | --- | --- | --- | --- | --- | --- | --- |
|  | All-cause mortality | | | CVD mortality | | | Non-CVD mortality | | |
|  | HR (95% CI) | | P-value | HR (95% CI) | | P-value | HR (95% CI) | | P-value |
| Measure | Per 1-unit increase | Per SD increase |  | Per 1-unit increase | Per SD increase |  | Per 1-unit increase | Per SD increase |  |
| Mean PPD, mm | 1.154 (1.023; 1.302) | 1.109 (1.017; 1.209) | 0.020 | 1.114 (0.901; 1.377) | 1.080 (0.927; 1.259) | 0.321 | 1.183 (1.023; 1.368) | 1.128 (1.017; 1.253) | 0.023 |
| Sites with PPD≥3mm, % | 1.005 (1.000; 1.009) | 1.127 (1.011; 1.255) | 0.031 | 1.002 (0.995; 1.010) | 1.057 (0.883; 1.264) | 0.547 | 1.007 (1.001; 1.012) | 1.176 (1.028; 1.345) | 0.018 |
| Sites with PPD≥4mm, % | 1.005 (1.000; 1.010) | 1.091 (1.003; 1.186) | 0.042 | 1.003 (0.995; 1.011) | 1.057 (0.917; 1.219) | 0.444 | 1.007 (1.000; 1.013) | 1.116 (1.008; 1.237) | 0.035 |
| Mean CAL, mm | 1.111 (1.053; 1.174) | 1.219 (1.101; 1.349) | <0.001 | 1.118 (1.020; 1.226) | 1.232 (1.038; 1.464) | 0.017 | 1.111 (1.039; 1.188) | 1.217 (1.074; 1.380) | 0.002 |
| Sites with CAL≥3mm, % | 1.009 (1.004; 1.013) | 1.351 (1.153; 1.584) | <0.001 | 1.005 (0.997; 1.014) | 1.205 (0.908; 1.598) | 0.196 | 1.010 (1.005; 1.016) | 1.437 (1.185; 1.741) | <0.001 |
| Sites with CAL≥4mm, % | 1.007 (1.004; 1.010) | 1.261 (1.127; 1.411) | <0.001 | 1.005 (0.999; 1.011) | 1.165 (0.964; 1.408) | 0.115 | 1.009 (1.005; 1.013) | 1.328 (1.155; 1.526) | <0.001 |
| Number of missing teeth | 1.024 (1.008; 1.040) | 1.173 (1.055; 1.305) | 0.003 | 1.028 (1.001; 1.056) | 1.210 (1.007; 1.454) | 0.042 | 1.021 (1.002; 1.041) | 1.154 (1.012; 1.316) | 0.032 |
| Periodontitis Score | - | 1.388 (1.184; 1.628) | <0.001 | - | 1.380 (1.041; 1.830) | 0.025 | - | 1.405 (1.157; 1.706) | 0.001 |
|  |  |  |  |  |  |  |  |  |  |
| **Systemic Inflammation ^**^** |  | | | | | | | | |
|  | All-cause mortality | | | CVD mortality | | | Non-CVD mortality | | |
|  | HR (95% CI) | | P-value | HR (95% CI) | | P-value | HR (95% CI) | | P-value |
| Measure | Per 1-unit increase | Per SD increase |  | Per 1-unit increase | Per SD increase |  | Per 1-unit increase | Per SD increase |  |
| Leukocytes, Gpt/l | 1.130 (1.079; 1.183) | 1.273 (1.162; 1.395) | <0.001 | 1.155 (1.063; 1.255) | 1.330 (1.128; 1.568) | 0.001 | 1.342 (1.140; 1.579) | 1.247 (1.117; 1.392) | <0.001 |
| Fibrinogen, g/l | 1.307 (1.143; 1.495) | 1.200 (1.095; 1.314) | <0.001 | 1.251 (0.986; 1.588) | 1.164 (0.990; 1.370) | 0.066 | 1.118 (1.057; 1.182) | 1.221 (1.093; 1.365) | <0.001 |
| CRP, mg/l, log-transformed | 1.201 (1.087; 1.326) | 1.231 (1.099; 1.379) | <0.001 | 1.207 (1.012; 1.441) | 1.239 (1.013; 1.515) | 0.037 | 1.195 (1.059; 1.349) | 1.225 (1.068; 1.406) | 0.004 |
| Inflammation score | - | 1.423 (1.257; 1.610) | <0.001 | - | 1.430 (1.148; 1.781) | 0.001 | - | 1.423 (1.224; 1.654) | <0.001 |

Effect estimates from Cox-proportional hazards models with classical time-invariant exposures and adjustments. A separate model was calculated for each exposure/mortality combination. Abbreviations: CVD, cardiovascular disease; PPD, pocket probing depth; CAL, clinical attachment level; CRP, C-reactive protein; HR, hazard ratio; CI, confidence interval; SD, standard deviation.

^*^ Models were adjusted for baseline values of age, sex, living in a partnership, tertiles of equivalised income, smoking status, categories of body mass index, diabetes mellitus, physical activity, tooth brushing frequency and regular dental check-ups.

^**^ Models were adjusted for baseline values of age, sex, living in a partnership, tertiles of equivalised income, smoking status, categories of body mass index, diabetes mellitus, physical activity, dyslipidaemia and high alcohol consumption.

**Table S5.** Interaction of periodontitis and systemic inflammation regarding mortality: overview and ranking.

| **Periodontitis measure** | **Systemic Inflammation measure** | | | |
| --- | --- | --- | --- | --- |
|  | Fibrinogen | Leukocytes | C-Reactive Protein | Inflammation score |
| Mean PPD | 00.158*0 | -0.003 | 0.181* | 000.200*^a^ 0 |
| Sites with PPD≥3mm | 00.138*0 | -0.049 | 0.1620 | 00.21900 |
| Sites with PPD≥4mm | 00.144** | 0.019 | 0.130* | 00.150** |
| Mean CAL | 00.08700 | 0.132 | **0.1480** | 00.20600 |
| Sites with CAL≥3mm | 00.007^a^0 | 0.123 | 0.175^a^ | 00.16800 |
| Sites with CAL≥4mm | 00.04600 | 0.113 | 0.1840 | 00.17400 |
| Number of missing teeth | 00.083^a^ 0 | 0.145 | 0.001* | 00.146^a^ 0 |
| Periodontitis Score | 00.110^a^ 0 | 00.233^a^ | 0.154^a^ | **00.27800** |

Synoptic overview of additive and multiplicative interaction measures for all combinations of variables considered, derived from Cox proportional hazards models with time-varying exposures and covariates.

The listed values represent the relative excess risks per SD increase due to interaction of the respective periodontitis (row) and systemic inflammation (column) measure with regard to CVD mortality (RERI_CVD_). Statistical significance (P<0.1) and also the proportion of effect sizes for additive interaction relative to mortality type are indicated via colour-coding. Statistical significance of corresponding multiplicative interaction measures and references to graphical or detailed numerical presentations are indicated by symbols.

Symbols and colours:

|  | Significance of at least RERI_­CVD_ and RERI_CVD_ > RERI_all-cause_ > RERI_non-CVD_ |
| --- | --- |
|  | No significance of RERI_­CVD_ but RERI_CVD_ > RERI_all-cause_ > RERI_non-CVD_ |
|  | Significance but RERI_CVD_ ≈ RERI_all-cause_ ≈ RERI_non-CVD_ |
|  | No significance, all RERI <0.05 and RERI_CVD_ ≈ RERI_all-cause_ ≈ RERI_non-CVD_ |
| * | Significance (P<0.1) of multiplicative interaction for CVD mortality only |
| ** | Significance (P<0.1) of multiplicative interaction for CVD mortality and all-cause mortality |
| **bold** | Main focus: Associated survival curves and crude IRR are presented in Figure 2, Figure 3 and Figure S2. Moreover, details on multiplicative and additive interaction terms as well as results from mediation analyses are presented in Table 3. |
| ^a^ | Associated survival curves and crude IRR are presented in Figure S3 and Figure S4. |

Abbreviations: CVD, cardiovascular disease; PPD, pocket probing depth; CAL, clinical attachment level; SD, standard deviation; IRR, incidence rate ratio; RERI, relative excess risk due to interaction; RERI_­CVD_ / RERI_all-cause_ / RERI_non-CVD_, additive interaction regarding CVD mortality / all-cause mortality / non-CVD mortality.

**Table S6.** Sample analytic code.

| **Objective** | **Code** |
| --- | --- |
|  | Single effects analyses with baseline adjustment |
| Defining confounder groups | global confounder_perio_bl male_bl age_bl i.income_3cat_bl partner_bl i.smoking_3cat_bl i.bmi_3kat_cat_bl diabetes_bl physact_bl denthyg_bl control_bl  global confounder_inflamm_bl male_bl age_bl i.income_3cat_bl partner_bl i.smoking_3cat_bl i.bmi_3kat_cat_bl diabetes_bl physact_bl dyslip_bl riskalc_bl |
| Setting data details regarding multi stage design and type of outcome, example for all-cause mortality | stset mort_time, failure(mort_all)  svyset psu [pweight=sample_weights], strata(STRATA1) fpc(pop_correction_values1) \|\| proband, strata(STRATA2) fpc(pop_correction_values2) |
| Analyses of exemplary periodotitis variables as exposures | xi: stcox pdm_bl $confounder_perio_bl, efron  xi: stcox avm_bl $confounder_perio_bl, efron  xi: stcox perio_score_bl $confounder_perio_bl, efron |
| Analyses of exemplary inflammation variables as exposures | xi: stcox wbc_bl $confounder_inflamm_bl, efron  xi: stcox log_crp_bl $confounder_inflamm_bl, efron  xi: stcox inf_score_bl $confounder_inflamm_bl, efron |
|  | Combined exposures & interaction analyses with baseline adjustment |
| Combined confounder group | global confounder_total_bl male_bl age_bl i.income_3cat_bl partner_bl i.smoking_3cat_bl i.bmi_3kat_cat_bl diabetes_bl physact_bl dyslip_bl riskalc_bl denthyg_bl control_bl |
| Combined exposures A & B | xi: stcox A B $confounder_total_bl, efron |
| Multiplicative interaction | xi: stcox A B AxB $confounder_total_bl, efron |
| Additive interaction | nlcom exp(_b[A]+_b[B]+_b[AxB])-exp(_b[A])-exp(_b[B])+1 |
|  | Single effects analyses with time varying exposures and confounders |
| Defining confounder groups | global confounder_perio male_tv age_tv i.income_3cat_tv partner_tv i.smoking_3cat_tv i.bmi_3kat_cat_tv diabetes_tv physact_tv denthyg_tv control_tv  global confounder_inflamm_tv male_tv age_tv i.income_3cat_tv partner_tv i.smoking_3cat_tv i.bmi_3kat_cat_tv diabetes_tv physact_tv dyslip_tv riskalc_tv |
| Setting data details regarding multi stage design, time interval specification and type of outcome, example for all-cause mortality | stset finish, enter(time start) failure(mort_all_tv) id(proband)  svyset psu [pweight= sample_weights], strata(STRATA1) fpc(pop_correction_values1) \|\| proband, strata(STRATA2) fpc(pop_correction_values2) |
| Analyses of exemplary time varying periodotitis variables as exposures | xi: stcox pdm_tv $confounder_perio_tv, efron  xi: stcox avm_tv $confounder_perio_tv, efron  xi: stcox perio_score_tv $confounder_perio_tv, efron |
| Analyses of exemplary time varying inflammation variables as exposures | xi: stcox wbc_tv $confounder_inflamm_tv, efron  xi: stcox log_crp_tv $confounder_inflamm_tv, efron  xi: stcox inf_score_tv $confounder_inflamm_tv, efron |
|  | Combined exposures & interaction analyses with time varying exposures and confounders |
| Combined confounder group | global confounder_total_tv male_tv age_tv i.income_3cat_tv partner_tv i.smoking_3cat_tv i.bmi_3kat_cat_tv diabetes_tv physact_tv dyslip_tv riskalc_tv denthyg_tv control_tv |
| Combined time varying exposures A & B | xi: stcox A B $confounder_total_tv, efron |
| Multiplicative interaction | xi: stcox A B AxB $confounder_total_tv, efron |
| Additive interaction | nlcom exp(_b[A]+_b[B]+_b[AxB])-exp(_b[A])-exp(_b[B])+1 |

The presented code is based on Stata notation.

Wherever possible, self-explanatory terms have been used. Colours have also been used for clarity.

All code examples function in theory but are specific to the dataset, data structure and certain background settings, not all of which can be displayed here.

Models with or without time-varying confounders primarily vary in terms of the data structure, as denoted by the suffixes used.

Suffixes:

_bl baseline

_tv time varying
